# Kinetic Analysis of HDAC‐ and Sirtuin‐Mediated Deacylation on Chemically Defined Histones and Nucleosomes

**DOI:** 10.1002/cpz1.70251

**Published:** 2025-11-08

**Authors:** Zhuoyi L. Niu, Ling Zhang, Yuan‐Fei Zhou, Zhipeng A. Wang

**Affiliations:** ^1^ Desai Sethi Urology Institute & Sylvester Comprehensive Cancer Center University of Miami Miller School of Medicine Miami Florida; ^2^ These authors contributed equally to this work

**Keywords:** deacylation, HDAC, histone protein, inhibitor, nucleosome core particle, sirtuin

## Abstract

Histone deacetylases (HDACs) and sirtuins (SIRTs) play essential roles in regulating chromatin structure and gene expression by catalyzing the removal of acyl groups from histone lysine residues. Accurate characterization of their deacetylation kinetics is critical for understanding their enzymatic mechanisms and for guiding inhibitor or activator development. Given the complexity of enzyme‐nucleosome core particle (NCP) interactions, including the influence of histone composition, post‐translational modifications, and DNA context, NCP‐based assays provide a more physiologically relevant platform than those performed on peptide or free histone substrates. Here, we present optimized protocols for assessing HDAC and SIRT deacetylation kinetics using NCP substrates, including determination of Michaelis‐Menten parameters, evaluation of inhibitor and activator potency (IC_50_ or EC_50_), and instructions for ensuring assay reproducibility. These methods enable robust comparison of small‐molecule modulators under conditions that better mimic the native chromatin environment, supporting both mechanistic studies and drug discovery efforts. © 2025 The Author(s). Current Protocols published by Wiley Periodicals LLC.

**Basic Protocol 1**: Assay of HDAC complex or SIRT deacylation on nucleosome substrates

**Basic Protocol 2**: Assay of HDAC complex or SIRT deacylation on free histone proteins

**Basic Protocol 3**: *K*_M_ measurement for deacylation assay on nucleosome or cofactor

**Basic Protocol 4**: Assessment of deacylation inhibitor or activator effects on NCP substrates

## INTRODUCTION

Protein post‐translational modifications (PTMs) can profoundly affect protein structure and function, and their dysregulation is implicated in diseases such as cancer (Dancy & Cole, 2015). Lysine acylation is one of the most abundant and diverse PTMs in mammalian cells, encompassing classical acetylation as well as more than 20 acylation types (Wang & Cole, 2020). Lysine acylation is finely and dynamically regulated by two groups of enzymes: histone acetyltransferases (HATs), also known as “writers,” which add acyl groups, and histone deacetylases (HDACs), also known as “erasers,” which remove them from lysine residues on both histone and non‐histone proteins (Jing & Lin, 2015). These enzymes function as essential epigenetic regulators of gene transcription and chromatin packaging.

**Figure 1:**
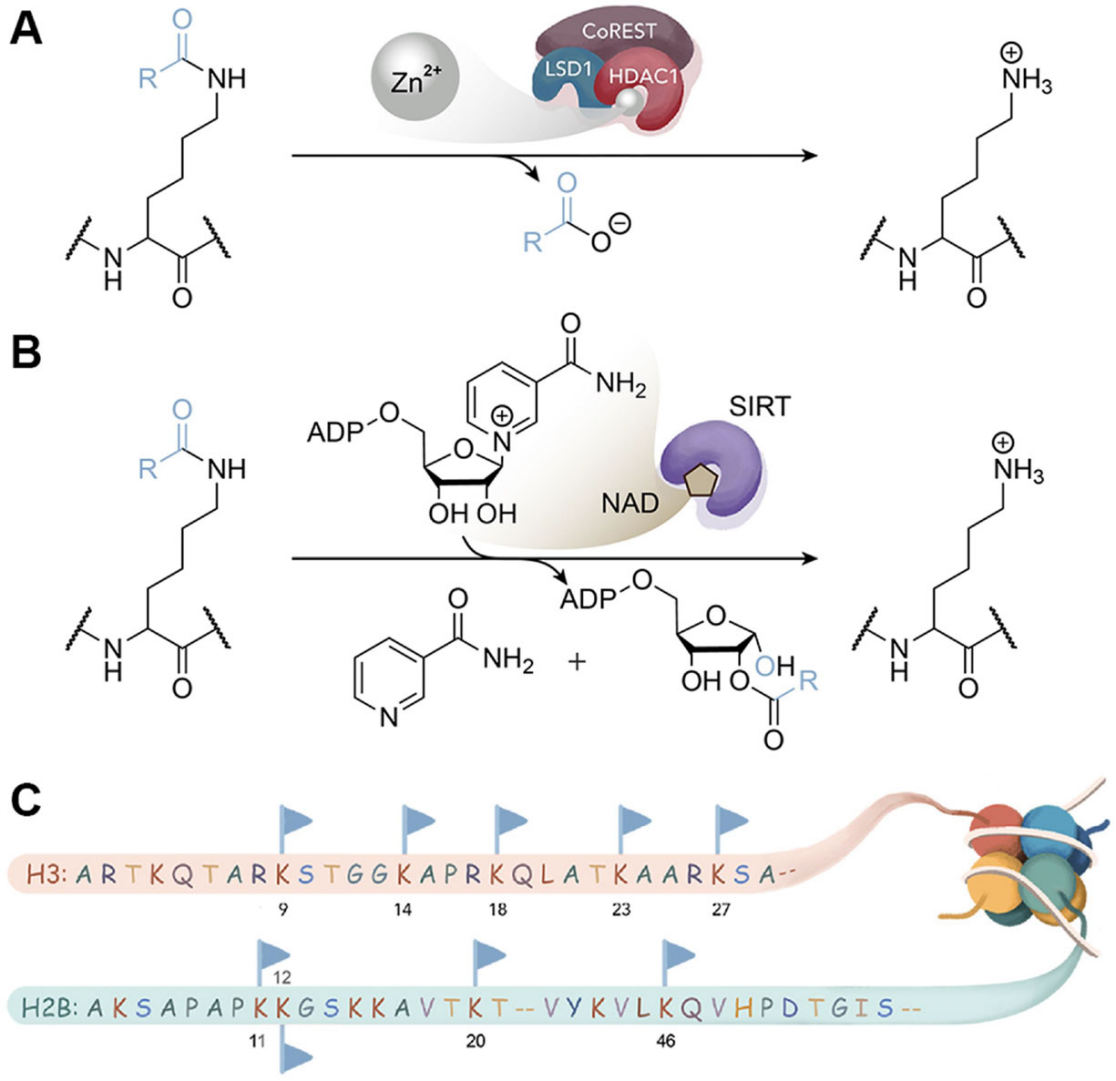
Mechanisms of HDAC complex and SIRT deacylation on representative nucleosomal substrates. (**A**) Catalytic mechanism of zinc‐dependent HDACs, exemplified by the CoREST complex containing HDAC1, LSD1, and CoREST. A Zn²⁺‐activated water molecule hydrolyzes the acyl‐lysine carbonyl group (R represents variable acyl groups), generating a deacylated lysine and releasing a carboxylate product. (**B**) Catalytic mechanism of NAD‐dependent SIRTs, in which NAD reacts with the acyl‐lysine to form 2′‐*O*‐acyl‐ADP‐ribose and nicotinamide, releasing the deacylated lysine. (**C**) Schematic representation of histone H3 and H2B N‐terminal tails within nucleosomal substrates, showing some common acylation sites (blue flags) that previously serve as substrates for deacylation assays.

HDACs are categorized into four classes, with classes I, II, and IV comprising HDAC1 to HDAC11. Although these 11 metallohydrolase HDACs share a conserved zinc‐dependent catalytic domain, they exhibit distinct subcellular localizations and biological functions. In particular, class I HDACs (HDAC1, HDAC2, HDAC3, and HDAC8), which account for the majority of deacetylase activity in cells (Kelly & Cowley, 2013), operate within distinct multiprotein complexes (Wang et al., 2020; Fig. 1A). The activity of these classes of HDACs is regulated by complex composition, subcellular localization, cell cycle timing, and other aspects of cellular state. In addition to the metallohydrolase HDACs, class III HDACs, known as sirtuins (SIRTs), comprise seven nicotinamide adenine dinucleotide (NAD)‐dependent enzymes that exhibit distinct subcellular localizations and specialized functions in human (Wang, Hsu, et al., 2017; Fig. 1B). This raises a longstanding question in the field: why do cells require such a large number of enzymes from two distinct families that perform similar deacetylase and deacylase activities?

Enzymology provides a valuable window into addressing this question by revealing the preferred protein substrates of each HDAC, the specific modification sites they target, and the rates at which deacetylation occurs (Wang et al., 2025). However, these questions are difficult to address using cell‐based assays due to the dynamic and transient nature of PTMs. For example, each protein, especially histones, often carries multiple, co‐existing modifications that can engage in crosstalk, and each PTM may be simultaneously regulated by multiple erasers (Whedon et al., 2024). Moreover, a single enzyme can act on multiple sites rather than a single target and may even exert competing effects on different acetylation sites within the same protein or protein complex (Abeywardana et al. 2024). These complexities make it nearly impossible to determine direct enzyme‐substrate interactions or to obtain clear kinetic parameters using conventional knockout or overexpression approaches. Some attempts to purify histone or nucleosome substrates and enzymes from cells for *in vitro* assays have helped simplify the system (You et al., 2019), but definitive analysis remains difficult because the combinatorial complexity of PTMs on the substrates is still not fully addressed.

Chemical biology, empowered by synthetic chemistry, enables the generation of chemically defined and pure substrates bearing a specific PTM at a defined site with 100% modification efficiency. Many *in vitro* enzymology studies rely on short synthetic peptide substrates because of their practicality and ease of chemical synthesis. These approaches have proven powerful for kinetic analysis in many systems. However, limitations remain. For example, peptide‐based assays often fail to fully recapitulate cellular observations (Feldman et al., 2013). A notable case is SIRT6, which efficiently deacetylates H3K9ac (Kawahara et al., 2009) and H3K18ac in cells (Tasselli et al., 2016) but shows little to no activity on corresponding peptide substrates *in vitro* (Wang et al., 2016).

With advances in chemical biology, including protein total synthesis, semi‐synthesis, bioorthogonal chemistry, and genetic codon expansion (Wang, 2019), PTMs can now be installed onto large proteins and protein complexes with high precision. This has enabled researchers to recognize that the structural complexity of chromatin and the spatial context of nucleosomes are far more sophisticated in regard to enzyme binding and orientation than can be modeled by short peptides (Lee et al., 2022). As a result, peptide‐based assays often fail to capture the biological relevance of chromatin‐modifying enzymes. These developments open new opportunities for studying writer, reader (which directly bind PTMs), and eraser complexes directly on nucleosomal substrates. However, performing kinetic analysis on modified nucleosomes is more challenging due to the high cost and precise preparation required to generate site‐specifically modified nucleosomes, as well as the dynamic nature of the nucleosome structure (Fig. 1C). This contrasts with short peptides, which are easier to characterize using techniques such as HPLC or mass spectrometry.

This protocol employs chemically defined nucleosomes containing site‐specifically modified full‐length histone proteins to guide experimental design and execution, using HDAC deacetylation assays as a primary example. This approach enables the measurement of HDAC and sirtuin activity in a highly physiologically relevant, though still *in vitro*, context (Liu et al., 2020). It also allows relatively accurate kinetic comparisons between enzyme and enzyme complexes, bridging traditional enzymology with chromatin‐based regulation. Furthermore, this strategy can be extended to assay development for other PTMs and other writer and eraser proteins. In addition, the development of inhibitors and activators would benefit from more structurally complex substrate conditions, rather than relying on unstructured peptide models (Wang et al., 2025).

## STRATEGIC PLANNING

### Enzyme and substrate preparation

Before conducting any of the following deacylation assay and kinetic experiments, it is essential to prepare HDAC complexes (Lee et al., 2025) or SIRT enzymes (Wang et al., 2023a), as well as full‐length free histone proteins or nucleosome core particles (NCPs) (Whedon et al., 2024). In general, class I HDAC complexes can be purified from mammalian cells, whereas tag‐free full‐length SIRTs are expressed in bacterial systems, and both should ideally be freshly prepared before use (Wang et al., 2022). Synthetic NCPs containing site‐specific modifications, whether single modifications, combinatorial patterns for crosstalk studies, or asymmetric modifications on the two H3 tails, can be prepared by total/semi‐synthesis or chemoenzymatic synthesis (Whedon et al., 2024).

Both HDAC complexes or SIRT enzymes and NCP substrates can be aliquoted and stored at –80°C at high concentration with 5%‐20% glycerol for weeks to months. Because of their dynamic nature, NCPs should be stored above 5 µM, avoiding freeze‐thaw cycles, and each aliquot should ideally be used within 1 week. HDAC complexes are also dynamic; after thawing, each aliquot should be used within 2 days, and prolonged storage at –80°C also significantly reduces enzymatic activity.

The assay is analyzed and quantified by Western blotting using site‐specific antibodies (see Nucleosome kinetic theory). Because Western blotting is a semi‐quantitative technique, different batches of HDAC complexes or SIRT enzymes or NCPs may yield variable results. Therefore, for parallel comparisons, whether testing different modifications with the same HDAC complex or SIRT or comparing different HDAC complexes or SIRTs on the same modified NCP, we recommend performing the assays as close together in time as possible.

### Pre‐characterization of site‐specific antibodies by Western blotting

As mentioned, Western blotting has been used to track and visualize the deacetylation activity of HDAC complexes and SIRTs with site‐specific anti‐acetyl‐lysine antibodies. This detection method generally provides strong affinity and allows accurate quantification of the remaining acetylated signal (Chio et al., 2023). This accuracy is particularly important because the goal is to monitor near‐complete removal of the acetyl mark to obtain a clear kinetic measurement (see Nucleosome kinetic theory).

Therefore, preliminary validation of antibody affinity and selectivity is essential. Five H3Kac‐site‐specific antibodies (anti‐H3K9ac, anti‐H3K14ac, anti‐H3K18ac, anti‐H3K23ac, and anti‐H3K27ac) have been validated, including comparisons on mono‐acetylated and penta‐acetylated H3 nucleosomes (Whedon et al., 2024). Similarly, four H2Bkac‐site‐specific antibodies (anti‐H2BK11ac, anti‐H2BK12ac, anti‐H2BK20ac, and anti‐H2BK46ac) have been tested (Wang et al., 2022). In previous studies, these antibodies showed nearly complete loss of signal after deacetylation, suggesting minimal nonspecific binding. For PTM‐eraser assays, it is our opinion loading controls are not essential because each time point is normalized to the band at time 0 (*t* = 0). Although some anti‐PTM antibodies may exhibit cross‐reactivity, this does not affect the results because the substrates used are chemically defined and pure.

Antibody linearity is critical for the quantitative measurement of Western blotting. A calibration curve should be generated to confirm that substrate recognition for all site‐specific antibodies (e.g., anti‐H3K9ac antibody) falls within the linear range. For example, different concentrations of H3K9ac nucleosome (0.025, 0.05, 0.1, 0.2, and 0.3 fmol) are loaded, and this is followed by primary and secondary antibody blotting and ECL detection to measure band intensity. The intensity is then plotted against the amount of H3K9ac nucleosome to determine the linear detection range. The results show good linearity between signal intensity and H3K9ac nucleosome input (Wang et al., 2023b).

### Deacetylation assay design for NCP

To conserve reagents, the assay volume is minimized to accommodate all time points within a 2‐hr period, with samples typically collected every 30 min. Sampling within the 0‐ to 30‐min window is not ideal and may hinder accurate measurement of deacetylation activity, because the reaction proceeds too slowly to produce a discernible decrease in Western blotting signal, which may compromise subsequent curve fitting during data analysis. Sampling beyond 2 hr carries the risk of enzyme inactivation and/or substrate degradation, which can compromise assay reliability.

For each assay, the final concentration of NCPs is standardized at 100 nM (see Nucleosome kinetic theory). However, enzyme concentrations vary significantly depending on their catalytic activity (e.g., 30 nM for CoREST, 5 nM for MiDAC). NCP concentrations are measured freshly using a NanoDrop spectrophotometer, and HDAC complex and SIRT concentrations are determined by SDS‐PAGE with Coomassie Blue staining, using a gradient of bovine serum albumin (BSA) as the standard. The goal is to identify an optimal kinetic window in which the enzymatic reaction follows a first‐order decay mechanism, ensuring reliable quantification while minimizing the risk of insufficient signal or enzyme denaturation over time.

For each assay, a final concentration of 0.2 mg/ml BSA is included, which helps prevent enzyme or NCP instability and reduces nonspecific adsorption to the tube walls, sometimes seen with very low protein concentrations. In cases where certain HDAC complexes (such as MiDAC) or SIRTs (such as SIRT6) act rapidly on preferred NCP substrates, the reaction may proceed too quickly. In such cases, rather than further decreasing the enzyme concentration, which could introduce substantial dilution error, sampling should be performed at multiple time points, typically between 0 and 60 min or 0 and 90 min. When working with a novel enzyme or an uncharacterized PTM, it is important to test at least two different enzyme concentrations to ensure linearity within appropriate enzyme concentration and kinetic ranges.

### Quench the protein samples at each time point before gel analysis

To terminate the enzymatic reaction at each time point, samples must be quenched to prevent continued activity. Standard SDS loading dye is used to halt the reaction; to ensure complete cessation, additional reagents are included. For HDACs, whose active sites coordinate Zn²⁺, EDTA is an effective reagent for chelating the metal ion and inactivating the enzyme. Therefore, we use a dual quenching (DQ) solution consisting of 2× Laemmli sample buffer supplemented with 20 mM EDTA. Although SIRTs do not rely on Zn²⁺ in their active sites, we still use DQ buffer because protein folding and activity often involve metal ions. Each reaction sample is quenched by adding an equal volume of DQ buffer to achieve final concentrations of 10 mM EDTA and 1× Laemmli buffer.

### Nucleosome kinetic theory

To evaluate different HDAC complexes on the same nucleosomal substrate, or to evaluate the same HDAC complex on different nucleosomal substrates, we value catalytic turnover number (*k*_cat_) as the most informative kinetic parameter. To estimate the *k*_cat_ for our nucleosome‐based enzymatic reactions, we assume that the system follows Michaelis‐Menten kinetics under the steady‐state approximation. This assumption is justified based on the slow catalytic step (*k*_2_) of general HDAC or HDAC‐complex‐catalyzed nucleosome deacylation, which is rate limiting (*k*_2_ ≪ *k*_‐1_), thereby supporting the steady‐state condition in which the concentration of the ES complex ([ES]) remains approximately constant.

$$V_{\max}=k_{\mathrm{cat}}\cdot \left[E_{t}\right]$$

Because of the limited availability and stability of nucleosome substrates, we have been unable to generate a full saturation curve to fit *V*_max_ and *K*_M_ directly in most cases. However, the apparent *K*_M_ values for the corresponding HDAC or HDAC complex interacting with nucleosomes are generally expected to be low (in the low nanomolar range), likely due to face‐to‐face surface complementarity and multiple noncovalent interactions that stabilize the enzyme‐nucleosome complex. Thus, we try to use substrate concentrations ([S]) well above the expected *K*_M_ of the corresponding HDAC or HDAC complex for nucleosome binding. This is actually confirmed by our report that *K*_M_ of SIRT6 for nucleosomes is ∼17‐19 nM, and we normally use [S] = 100 nM, ensuring near‐complete saturation of the enzyme (i.e., [ES] ≈ [E_t_], where t refers to total enzyme).

Under this saturating condition, the initial velocity (*V*_0_) at a single substrate concentration condition approaches the maximum velocity:

$$k_{\mathrm{cat}}=\frac{V_{\max}}{\left[E_{t}\right]}≅\frac{V_{0}}{\left[E_{t}\right]}$$

We are unable to directly capture initial velocities under our current experimental conditions for several practical reasons. First, nucleosomes are structurally unstable and not amenable to rapid kinetic techniques such as stopped‐flow or continuous‐mix assays. Second, unlike for small‐molecule substrates or peptide‐based reactions, there is no suitable fluorometric or spectrophotometric method to continuously monitor nucleosome deacetylation in real time. As a result, we rely on Western blotting as our primary detection method. However, Western blotting is inherently an endpoint and discontinuous assay, typically requiring time intervals of at least 10‐30 min between sample collections. This makes it impractical to accurately measure the initial linear portion of the reaction where the substrate concentration remains effectively constant. In addition, Western blotting is only semi‐quantitative; to obtain meaningful signal differences, we often aim for 50%‐90% conversion of the acetylated nucleosome substrate. This requirement pushes the measurement further away from the true initial rate regime. High‐performance liquid chromatography (HPLC) is widely used in peptide and protein chemistry for kinetic studies. However, in our case, the reaction involves the removal of a single acetyl group from a nucleosomal histone protein over 100 amino acids in length, which poses a significant challenge for separation by conventional reverse‐phase HPLC. The minimal mass change and the lack of substantial polarity differences between the acetylated and deacetylated forms often result in poor chromatographic resolution. LC‐MS‐based strategies have been explored and are conceptually appealing, offering site‐specific detection. However, in our hands, these approaches have suffered from poor reproducibility and batch‐to‐batch variability, making them less reliable for robust kinetic quantification. Thus, given the lack of real‐time or high‐throughput alternatives for nucleosome substrates, Western blotting remains the only viable strategy to estimate enzymatic kinetics in this system.

We then adopt a pseudo‐first‐order kinetic model to extract an apparent rate constant from time‐course data from Western blotting (where *t* refers to time). Again, using SIRT6 as example, assuming that [NAD] and [E] are constant and [NAD] is in excess during the reaction (in the micromolar range), then the reaction simplifies to a first‐order process with respect to [S]:

$$V_{t}=\frac{d\left[S\right]}{dt}=-k\left[E\right]\left[S\right]\left[\mathrm{NAD}\right]=-k_{\mathrm{obs}}\left[S\right]$$

Integration of this differential equation yields an exponential decay function for substrate (or signal) loss:

$${\left[S\right]}_{t}={\left[S\right]}_{0}\cdot e^{-k_{\mathrm{obs}}\cdot t}$$

i.e.:

$$\frac{{\left[S\right]}_{t}}{{\left[S\right]}_{0}}=e^{-k_{\mathrm{obs}}\cdot t}$$

In practice, we monitor substrate disappearance or product accumulation using western blot band intensity over time. Intensities are normalized to the initial band intensity signal (*I*_0_), and the resulting dimensionless data are fit to an exponential decay function:

$$\frac{I_{\mathrm{remaining}}}{I_{0}}=\frac{I_{0}-I_{t}}{I_{0}}=1-e^{-k_{\mathrm{obs}}\cdot t}$$

Using GraphPad Prism “One phase decay” fitting (https://www.graphpad.com/guides/prism/latest/curve‐fitting/reg_exponential_decay_1phase.htm), we can get the fitted *K* value for the exponential decay. The *k*_obs_ value (in min^−1^) is:

$$k_{\mathrm{obs}}≅K$$

It is important to note that as the substrate is consumed over the course of the reaction, the first‐order decay constant (*k*_obs_) itself is not truly constant, especially when [S] is no longer ≫*K*_M_. Therefore, the *k*_obs_ value derived from exponential fitting represents an average rate constant across the entire reaction window, typically spanning 1‐2 hr. This approximation is especially relevant in endpoint assays such as Western blotting, where continuous monitoring is not feasible. Based on this averaged *k*_obs_, we estimate an apparent initial velocity under pseudo‐steady‐state conditions as follows: maximum velocity:

$$V_{0}^{\mathrm{app}}=k_{\mathrm{obs}}\cdot {\left[S\right]}_{0}=K\cdot {\left[S\right]}_{0}≅V_{\max}^{\mathrm{app}}$$

and subsequently calculate the apparent turnover number:

$$k_{\mathrm{cat}}^{\mathrm{app}}=\frac{V_{\max}^{\mathrm{app}}}{\left[E_{t}\right]}≅\frac{V_{0}^{\mathrm{app}}}{\left[E_{t}\right]}=\frac{K\cdot {\left[S\right]}_{0}}{\left[E_{t}\right]}$$

And thus, we simplify it as:

$$k_{\mathrm{cat}}^{\mathrm{app}}=\frac{V}{\left[E\right]}$$

This strategy assumes that the substrate is saturating, the enzyme is fully active, and the exponential fitting captures the overall reaction rate in the pseudo‐steady‐state regime. In cases where multiple enzyme concentrations are tested, a linear correlation between *k*_obs_ and [E_t_] is expected, serving as an internal validation for model assumptions. A non‐linear relationship would indicate deviation from simple Michaelis‐Menten behavior and suggest a more complex catalytic mechanism.

For estimating the total enzyme concentration [E_t_], we typically rely on UV absorbance at 280 nm or BCA/Bradford assays using a plate reader, calibrated with BSA standards. However, for HDAC complexes or SIRT family proteins, it is often difficult to guarantee the purity of the preparation because of co‐purified components, post‐translational heterogeneity, and/or incomplete processing. Therefore, we estimate the concentration of the catalytically active HDAC or SIRT enzyme by SDS‐PAGE, using a BSA standard curve to quantitatively compare band intensity with that of the purified target protein.

**Figure 2.**
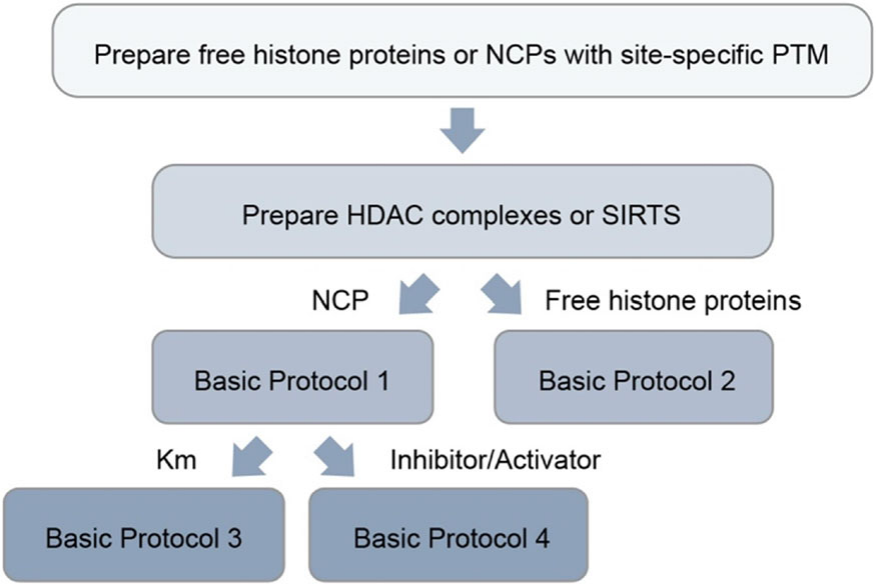
Workflow of the general protocols. Free histone proteins or NCPs carrying site‐specific PTMs are prepared as substrates (not discussed here; Whedon et al., 2024), and this is followed by the preparation of HDAC complexes (Lee et al., 2025) or SIRTs (Abeywardana et al., 2024; not discussed here) for deacetylation assays on NCP (Basic Protocol 1) or free histone proteins (Basic Protocol 2). Downstream analyses include Michaelis‐Menten kinetics for *K*_M_ determination (Basic Protocol 3) and inhibitor/activator assays (Basic Protocol 4).

The measurement of *K*_M_ in Michaelis‐Menten kinetics, as well as the assessment of inhibitors or activators, are discussed in the protocols below (Fig. 2). The calculations and plotting, however, follow the same principles as traditional single protein or enzyme assays and are therefore not described here in detail.

## Basic Protocol 1 ASSAY OF HDAC COMPLEX OR SIRT DEACYLATION ON NUCLEOSOME SUBSTRATES

### Materials

HDAC1 complexes: e.g., CoREST (HDAC1), MiDAC (HDAC1), and SMRT (HDAC3), ∼1‐10 µM stock solution (homemade)
Bovine serum albumin (BSA)
TBST (see recipe)
Coomassie blue stain
NCP with site‐specific PTM, ∼5‐10 µM stock solution (homemade)
Reagents and solutions for preparation of 1× HDAC reaction buffer (see recipe) or 1× SIRT reaction buffer (see recipe), as applicable
Reagents and solutions for preparation of DQ buffer (see recipe)
SIRT1, SIRT2, SIRT3, SIRT5, or SIRT6 (WT, mutant, or truncated forms), 10‐100 µM stock solution (homemade)
TBE buffer (see recipe)
SDS running buffer (see recipe)
Protein markers (Bio‐Rad, cat. no. 1610374)
Site‐specific anti‐acetyl/acyl‐lysine antibodies, as anti‐PTM (e.g., anti‐H3K9ac, Abcam, cat. no. ab32129)
Total histone antibody (e.g., anti‐H3, Abcam, cat. no. ab1791)
ECL solution (Bio‐Rad, cat. no. 170‐5061)
DNA ladder: e.g., Trackit 100 bp (Invitrogen, cat. no. 10488058)
Ethidium bromide solution (Sigma, cat. no. E1510‐10ML)

NanoDrop spectrophotometer (Thermo Fisher) or equivalent
Native gel electrophoresis system: e.g., 4%‐20% TBE gels from Novex (Thermo Fisher Scientific EC62252BOX) or equivalent
SDS‐PAGE gel electrophoresis system and Western blot transfer apparatus (e.g., Bio‐Rad or equivalent)
Gel imaging system capable of detecting chemiluminescence‐, fluorescence‐, and Coomassie‐stained SDS‐PAGE gels (Bio‐Rad ChemiDoc)
ImageJ software
Incubator, set at 37°C
GraphPad Prism software
Pipets and sterile filter tips
Microcentrifuge
Ice bucket

1 Just before setting up the assay, determine the concentrations of HDAC complexes or SIRTs in the samples using SDS‐PAGE followed by Coomassie blue staining, with a gradient of BSA as the standard for quantification.
  A linear standard curve is generated using BSA in the range of 0.1 mg/ml to 2.0 mg/ml, typically with 5‐8 concentration points. Concentrations above this range may result in reduced linearity. Multiple dilutions of the HDAC complex are run to ensure that at least one sample falls within the linear range of the BSA curve. Quantification is performed based solely on the HDAC band (e.g., full‐length HDAC1, amino acids 1‐482, ~55 kDa) or SIRT band (e.g., SIRT6, amino acids 1‐355, ~39 kDa).
2 Measure NCP concentrations using a NanoDrop spectrophotometer.
  All frozen stock samples stored at –80°C should be thawed slowly on ice. Mix briefly by gentle pipetting up and down 3‐4 times, then briefly centrifuge at ∼5000 rcf, 4°C, using a “fast” spin program. Do not vortex or invert the tubes to avoid disrupting the sample integrity.
  The concentration of NCP is calculated based on DNA content, as each nucleosome contains one DNA molecule. The molecular weight (MW) of 147 bp DNA is 90,874.63 Da. For example, if the DNA concentration (C_DNA_) measured by NanoDrop is 463.3 ng/µl, then the NCP concentration is calculated as:
$$\left[\mathrm{NCP}\right]=\frac{C_{\mathrm{DNA}}}{MW_{\mathrm{DNA}}}=\frac{463.3\;\mathrm{ng}/\mu L}{90,874.63\;g/\mathrm{mol}}\times \;1000=5.1\;\mu M$$
3 Plan the assay based on the freshly measured concentrations.
  Once we obtain the concentrations of HDAC complexes or SIRTs and NCPs, it is important to plan the reaction carefully and calculate all required components. For a standard 100 nM NCP assay, we will load 5 µl of a 1:1 sample‐to‐DQ mixture per time point into each SDS‐PAGE gel lane. This volume ensures clear Western blot signals and reliable quantification. The necessary components will be calculated as shown in the tables below.
  In this example, we demonstrate the dilution of H3K9ac‐NCP to a final working concentration of 1 µM (Table 1) and the dilution of SIRT6 to a final concentration of 200 nM (Table 2). As an illustration, the SIRT6 deacetylation assay using H3K9ac‐NCP as substrate is performed at two enzyme concentrations, 10 nM and 30 nM, to ensure a linear response versus enzyme concentration (Table 3). For each concentration, samples should be prepared and run in duplicate simultaneously. Duplicates of each concentration will be loaded on the same gel to ensure consistency across replicates. For each assay, samples will be collected at 0, 30, 60, 90, and 120 min. Therefore, the reaction mix is calculated and prepared in sufficient volume to accommodate sampling at more than five time points (Table 3). The final NAD concentration in this assay is 1.139 mM, which is well above the measured K_M_ for NAD (140 ± 19 µM); thus, the slight deviation from 1.00 mM does not affect the outcome. The design and calculations for the HDAC complex assay follow the same principles and are therefore not included here.

**Table 1:** Example of H3K9ac‐NCP Dilution to a Final Working Stock of 1 µM

Reagent	Stock	Working stock	Volume
H3K9ac‐NCP	6.05 µM	1.0 µM	2.8 µl
1× HDAC or SIRT reaction buffer			14.2 µl
Total			17.0 µl

**Table 2:** Example of SIRT6 Dilution to a Final Working Stock of 200 nM

**Reagent**	**Stock**	**Working stock E1**	**Volume**
SIRT6	41.3 µM	4.13 µM	3.0 µl
1× SIRT reaction buffer			27.0 µl
Total			30.0 µl
**Reagent**	**Working stock E1**	**Working stock E2**	**Volume**
SIRT6	4.13 µM	200 nM	2.0 µl
1× SIRT reaction buffer			39.3 µl
Total			41.3 µl

**Table 3:** Example Setup of a SIRT6 Deacetylation Assay on H3K9ac‐NCP in Duplicate for Two Concentrations of SIRT6

	SIRT6, 10 nM		SIRT6, 30 nM	
Reagent	Rep1	Rep2	Rep1	Rep2
1× SIRT reaction buffer	34 µl	34 µl	30 µl	30 µl
1 µM NCP	4.0 µl	4.0 µl	4.0 µl	4.0 µl
200 nM SIRT6	2.0 µl	2.0 µl	6.0 µl	6.0 µl
Total	40 µl	40 µl	40 µl	40 µl

4 Freshly prepare 1 ml total of the appropriate 1× reaction buffer—1× HDAC reaction buffer for HDAC complexes (Table 4) or 1× SIRT reaction buffer for SIRTs (Table 5)—for all subsequent dilutions. After adding all components, mix thoroughly by gentle pipetting up and down 6‐8 times using a 1‐ml pipet. Then briefly centrifuge the mixture at ∼5000 rcf, 4°C, using a “fast” spin program.
  Before these solutions are prepared, a 2 mg/ml BSA stock solution should be prepared, aliquoted, and stored at –80°C. A 10 mM dithiothreitol (DTT) stock solution can also be prepared, aliquoted, and stored up to 1 month at –80°C. When preparing the 1× reaction buffer, always add water first, followed by other components in order from largest to smallest volume. HEPES buffer is stored at room temperature but must be confirmed to be at pH 7.5 (ion strength = ∼‐69 mV) for each batch of final 1× reaction buffer after setup.
  When mixing solutions, always use a pipet volume larger than half of the total solution volume to ensure thorough mixing by pipetting up and down. This may require changing to a larger pipet after adding a component.

**Table 4:** Example of the Preparation of 1× HDAC Reaction Buffer

Reagent	Stock	Final	Volume
HEPES, pH 7.5	500 mM	50 mM	100 µl
KCl	2.0 M	100 mM	50 µl
BSA	2.0 mg/ml	0.2 mg/ml	100 µl
IP6	2.0 mM	0.1 mM	50 µl
H_2_O	–		700 µl
Total			1000 µl

**Table 5:** Example of the Preparation of 1× SIRT Reaction Buffer

Reagent	Stock	Final	Volume
HEPES, pH 7.5	500 mM	50 mM	100 µl
DTT	10 mM	1.0 mM	100 µl
BSA	2.0 mg/ml	0.2 mg/ml	100 µl
NAD	56.95 mM	1.0 mM	17.6 µl
H_2_O	–		772.4 µl
Total			1000 µl

5 Prepare DQ buffer (see recipe) and aliquot according to the assay design.
  Prepare the DQ solution by mixing equal volumes of 40 mM EDTA and 4× Laemmli sample buffer (either commercially available 4× Laemmli buffer or homemade 4× SDS loading buffer). Mix thoroughly by vortexing, then briefly centrifuge at ∼5000 rcf, 4°C, using a quick spin setting. Due to the viscous nature of 4× SDS loading buffer, accurate pipetting can be challenging. Pipet slowly to improve accuracy. Minor deviations are acceptable because all‐time points are normalized to the t = 0 sample and use the same DQ buffer batch.
  During pipetting, always touch the outer edge of the pipet tip to the tube wall when aspirating to remove any residual liquid from the tip exterior, minimizing unintended volume addition. When dispensing, press firmly to the second stop of the pipet to ensure complete delivery of the solution. Do not reuse pipet tips, even for the same reagent (e.g., DQ buffer), to maintain consistency and avoid contamination.
6 Prepare a 1 µM working stock solution of NCP in 1× reaction buffer as planned in Table 1. Mix thoroughly, centrifuge briefly, and keep on ice until use.
  The stock concentrations of NCP and HDAC complex may vary (containing Tris or any other buffer, with or without glycerol), but this does not generally affect the reaction outcome because their contributions to the final reaction volume appear to be negligible.
7 Prepare the assay by diluting NCP to a final concentration of 100 nM in 1× reaction buffer (Table 3). Mix briefly, centrifuge briefly, and keep the solution on ice until use.
  Because 100 nM NCP remains stable at 37°C for >2 hr, always dilute NCP into the reaction buffer first, rather than diluting the enzyme, to preserve maximum enzymatic activity. This is especially important when performing multiple assays in parallel to compare reaction rates. Based on our experience, no more than 12 assays should be set up simultaneously. Because both NCP and enzyme samples are precious, using two technical replicates per condition is acceptable if they yield consistent data and demonstrate clear linearity with respect to enzyme concentration. This allows up to six different conditions to be compared in a single experimental batch.
8 Dilute the HDAC complexes or SIRTs to the desired concentration in 1× reaction buffer as planned in Table 2. Mix thoroughly, centrifuge briefly, and keep the solution on ice until use.
  If the original stock concentration of the enzyme is too high, a two‐step dilution may be necessary. This is because adding a very small volume of highly concentrated enzyme into a much larger volume can lead to poor mixing and inconsistent results. From our experience, it is best to avoid dilutions exceeding 50‐fold in a single step (e.g., do not add less than 2 µl into 100 µl). If a multi‐step dilution is required, be sure to mix thoroughly and briefly centrifuge after each dilution step to ensure homogeneity.
  In quantitative enzymology and kinetic studies, avoid using pipet volumes <1 µl, and ideally use volumes >2 µl. Even with a well‐calibrated pipet, measurement errors at very low volumes are relatively high and can significantly affect data accuracy. To minimize variability, plan your calculations ahead of time to ensure that all additions can be made with adequately measurable volumes.
9 Add the HDAC complexes or SIRTs from step 8 to the desired final concentration in the reaction tube from step 7, as described in Table 3. Mix thoroughly, centrifuge briefly, and keep the solution on ice until use.
  When mixing the final reaction solution, always use a pipet with a volume capacity greater than half of the total solution volume—ideally close to the full volume—to ensure thorough mixing by pipetting up and down.
10 Take the *t* = 0 sample by mixing 1:1 with DQ buffer; in this example, 5 µl of sample is mixed with 5 µl of DQ buffer. Heat at 95°C for ∼1 min to quench and then store the sample on ice.
  Although literature protocols typically define the t = 0 sample as a no‐enzyme control, we have found that omitting the enzyme leads to uneven Western blot band intensities that are unreliable for normalization. To address this, we take the t = 0 sample immediately after adding the enzyme. This adjustment introduces minimal error because the reaction mixture is kept on ice prior to incubation, significantly suppressing enzymatic activity, and the quenching is performed rapidly—usually within 1 min. Under these conditions, little to no substrate conversion occurs, especially because the HDAC complexes and SIRTs used in our assays require extended incubation (>30 min) to produce a measurable change in band intensity.
11 Incubate the reaction at 37°C and collect samples at 30, 60, 90, and 120 min. Quench each time point by mixing 1:1 with DQ buffer and boiling briefly (for ∼1 min), and then store on ice.
  Avoid using a heating block when possible, as it only heats the bottom of the tube, leaving the lid unheated. During a 2‐hr incubation at 37°C, this can lead to condensation on the lid. Instead, use a 37°C incubator. Although this requires moving the entire rack out from incubator to collect samples, doing so within 1 min is acceptable. No shaking or stirring is needed during incubation.
  Because the reaction mixture is already well mixed, additional pipetting is not necessary when taking samples. Similarly, when adding samples to the DQ buffer, pipetting is not required, as the subsequent 95°C heating step will suffice.
12 Boil all samples at 95°C for 5 min and then centrifuge at ≥16,000 rcf, 25°C, for 3 min. Load the supernatant (5 µl for each sample, in this example) onto an SDS‐PAGE gel and run at 180 V for 20‐25 min.
  A gradient gel is recommended for optimal resolution of histones, though homemade 12% or 15% gels are also acceptable. If the gel is to be run the next day or re‐run for Western blotting because of suboptimal results, samples may be stored at 4°C overnight; however, long‐term storage is not recommended. Before reuse for gel analysis, reheat samples at 95°C for 5 min after overnight storage.
  Five time‐point samples from replicate 1 and five from replicate 2 should be run side by side on a single 15‐well gel. The two sets of replicate samples should be separated by a protein marker lane (Bio‐Rad, cat. no. 1610374). To minimize potential inconsistencies in Western blot running or transfer, the first and last lanes of the gel should not be used.
13 Excise the gel regions above 25 kDa and below 10 kDa to retain only the 10‐ to 25‐kDa region for membrane transfer.
  We typically use the iBlot transfer system with “P3” (20 V for 7 min), but other semi‐dry or wet transfer methods are also acceptable. Be sure to optimize transfer conditions, as histones are small proteins and may pass through the membrane. Both PVDF and nitrocellulose membranes are suitable.
  When comparing different enzyme concentrations or evaluating various HDAC complexes or SIRTs on the same substrate, it is advisable to transfer multiple gels onto a single membrane for direct comparison. Use distinct molecular weight marker patterns to distinguish each gel after transfer. Do not rely solely on activity‐dependent bands for lane identification, as low enzymatic activity or variability may result in undetectable or misleading signals—including apparent increases. Clear and consistent labeling, especially for the t = 0 time point, is critical for accurate interpretation. Marker patterns (e.g., lane position, number of bands, band intensities) can be used to differentiate gels or indicate orientation. Avoid overloading the marker, as this may suppress nearby protein signals; if possible, leave an adjacent empty lane to prevent interference with real signals.
14 Prepare 5% BSA in 1× TBST, either freshly or stored at 4°C for up to 1 week. Block the membrane by incubating in 5% BSA in 1× TBST for 1 hr at room temperature. Rinse briefly with 1× TBST.
15 Dilute the anti‐PTM primary antibody in 5% BSA in 1× TBST (typically 1:2000‐1:5000) and incubate overnight at 4°C. In parallel, incubate the membranes to be used for loading control with an anti‐histone antibody, i.e., H2B, H3, or H4, depending on the specific histone carrying the modification.
  A separate gel should always be run for anti‐histone Western blotting as a loading control. Stripping and re‐probing is not recommended, as it typically fails to completely remove the initial antibody signal. In NCP‐based assays, the anti‐histone signal generally remains stable over time. Therefore, to conserve material, it is not necessary to run multiple replicates solely for the anti‐histone blot. As membranes are not stripped, we do not normalize individual time points to the anti‐histone signal during data quantification.
16 The following day, wash the membrane with 1× TBST and then incubate with the appropriate secondary antibody at room temperature for 1 hr.
17 After washing the membrane, apply ECL solution and visualize the signal using an imager.
  For antibodies with good affinity and high‐quality nucleosomes, a low‐sensitivity ECL reagent is typically sufficient to yield clear bands. However, if the signal‐to‐noise ratio is poor, several parameters may need to be optimized—including exposure time, use of high‐sensitivity ECL reagents, and pre‐incubation time with the ECL solution. There is no universal optimal condition for Western blotting, especially for anti‐PTM antibodies. Each experiment may require optimization.
18 Collect gel image, quantify bands using ImageJ, and perform calculations using GraphPad Prism (see Understanding Results). A one‐phase decay curve (*Y*_0_ = 1) should be used to determine the fitting constant *K* for subsequent *V*/[E] value calculations.
  ImageJ band quantification requires careful handling, especially when signals are weak or background noise is high. In such cases, manually drawing tangent baselines to define peak areas is necessary for accurate measurement.
19 For the remaining samples in the assay tube, run a native gel (120 V, 120 min) in 0.5× TBE buffer in parallel with the SDS‐PAGE to confirm that the NCP remains intact, using a DNA ladder such as Trackit 100 bp (Invitrogen, cat. no. 10488058). Include a small amount of untreated NCP as a positive control. Stain with ethidium bromide and visualize using an imager.

## Basic Protocol 2 ASSAY OF HDAC COMPLEX OR SIRT DEACYLATION ON FREE HISTONE PROTEINS

### Materials (also see Basic Protocol 1)

HDAC1 complexes: e.g., CoREST (HDAC1), MiDAC (HDAC1), and SMRT (HDAC3), ∼1‐10 µM stock solution (homemade); *or* SIRT1, SIRT2, SIRT3, SIRT5, or SIRT6 (WT, mutant, or truncated forms), 10‐100 µM stock solution (homemade)
Coomassie blue stain
Free histone proteins (H3 or H2B) with site‐specific PTM from total or semisynthesis, or genetic codon expansion (Wang, Kurra, et al., 2017), ∼100‐200 µM stock solution (free peptide left over from synthesis or ligation should be completely removed by HPLC, as they may serve as better substrates for HDAC complexes or SIRTs than the full‐length proteins)
Site‐specific anti‐acetyl/acyl‐lysine antibodies (e.g., anti‐PTM)
Total histone antibody (e.g., anti‐H3, Abcam, cat. no. ab1791)

SDS‐PAGE gel electrophoresis system and Western blot transfer apparatus (e.g., Bio‐Rad or equivalent)
Gel imaging system capable of detecting chemiluminescence and Coomassie‐stained SDS‐PAGE gels (Bio‐Rad ChemiDoc)
Pipets and sterile filter tips
Microcentrifuge
Ice bucket
Heating block
Incubator, set at 37°C
GraphPad Prism
ImageJ

1 Just before setting up the assay, determine the concentrations of HDAC complexes or SIRTs in the samples using SDS‐PAGE followed by Coomassie Blue staining, with a gradient of BSA as the standard for quantification, as described in Basic Protocol 1, step 1.
2 Measure the concentrations of free histone proteins using SDS‐PAGE followed by Coomassie Blue staining, with a gradient of BSA as the standard for quantification.
  Unlike NCP frozen stocks, which are stored at –80°C, free histone proteins are stored as lyophilized powders. Before lyophilization, each tube should be aliquoted to ∼0.7‐1 mg. Although pure water does not fully dissolve histones, it is used to prepare assay‐compatible buffer conditions, and histones are known to refold upon dissolution in water in a different and heterogeneous state compared with the histone octamer. To reconstitute, add ∼1 ml of water to obtain a ∼100 µM stock solution. Mix thoroughly by pipetting up and down 6‐8 times with a 1‐ml pipet and then briefly centrifuge at ∼5000 rcf, 4 °C, using a “fast” spin program. Do not vortex or invert the tube, as this may cause histone precipitation. The concentration of site‐specifically modified histone proteins is calculated based on their theoretical molecular weights and confirmed by ESI or MALDI after preparation.
3 Plan the assay based on the freshly measured concentrations.
  Once you obtain the concentrations of HDAC complexes or SIRTs and histone proteins, it is important to plan the reaction carefully and calculate all required components. The necessary components will be calculated as shown in the tables below.
  In this example, we demonstrate the dilution of H3K9ac‐protein to a final working concentration of 10 µM (Table 6) and the dilution of SIRT6 to a final concentration of 3.0 µM (Table 7). As an illustration, the SIRT6 deacetylation assay using H3K9ac‐protein is performed at the maximum enzyme concentration of 300 nM (Table 8). The design and calculations for the HDAC complex assay follow the same principles and are therefore omitted.

**Table 6:** Example of H3K9ac‐protein Dilution to a Final Working Stock of 10 µM

Reagent	Stock	Working stock	Volume
H3K9ac‐protein	62.6 µM	10 µM	4.3 µl
1× HDAC or SIRT reaction buffer			22.6 µl
Total			26.9 µl

**Table 7:** Example of SIRT6 Dilution to a Final Working Stock of 3 µM

Reagent	Stock	Final	Volume
SIRT6	41.3 µM	3.0 µM	2.0 µl
1× SIRT reaction buffer			25.5 µl
Total			27.5 µl

**Table 8:** Example Setup of a SIRT6 Deacetylation Assay on H3K9ac‐protein in Duplicate

Reagent	Rep1	Rep2
1× SIRT reaction buffer	32 µl	32 µl
10 µM protein	4.0 µl	4.0 µl
3 µM SIRT6	4.0 µl	4.0 µl
Total	40 µl	40 µl

4 Freshly prepare 1 ml total of 1× reaction buffer—1× HDAC reaction buffer for HDAC complexes or 1× SIRT reaction buffer for SIRTs—for all subsequent dilutions as described in Basic Protocol 1, step 4.
5 Prepare DQ buffer and aliquot according to the assay design as described in Basic Protocol 1, step 5.
6 Prepare a 10 µM working stock solution of free histone protein in 1× reaction buffer (Table 6). Mix thoroughly, centrifuge briefly, and keep on ice until use.
7 Prepare the assay by diluting free histone protein to a final concentration of 1 µM in 1× reaction buffer. Mix briefly, centrifuge briefly, and keep the solution on ice until use.
  Overall, free histone proteins are less stable and reproducible than NCPs, making histone‐based assays less definitive and informative than the NCP assay in Basic Protocol 1. To minimize variability, comparisons between different enzymes should be performed using the same batch of histone stock. Reaction times should be kept shorter, as substantial reductions in histone band intensity are often observed in the anti‐histone control. No more than eight assays should be conducted simultaneously under identical conditions. Given that both semisynthetic histone proteins and enzyme samples are precious, using two technical replicates per condition is generally sufficient if they yield consistent results and exhibit clear linearity with respect to enzyme concentration. This setup allows up to four different conditions to be assessed in a single experimental batch.
8 Dilute the HDAC complexes or SIRTs to the desired concentration according to the experimental design as working stock solution in 1× reaction buffer (Table 7); and then mix thoroughly, centrifuge briefly, and keep the solution on ice until use (as described in Basic Protocol 1, step 8).
9 Add the HDAC complexes or SIRTs to the desired final concentration in the reaction tube (Table 8); and then mix thoroughly, centrifuge briefly, and keep the solution on ice until use (as described in Basic Protocol 1, step 9).
10 Take the *t* = 0 sample by mixing 1:1 with DQ buffer, heat at 95°C for ∼1 min to quench, and store the sample on ice, as described in Basic Protocol 1, step 10.
11 Incubate the reaction at 37°C and collect samples at 5, 10, 20, and 30 min. Quench each time point by mixing 1:1 with DQ buffer, briefly boiling for ∼1 min, and then storing on ice.
  Unlike the NCP assay, the free histone protein assay is better performed using a heating block due to the short incubation time. The block heats only the bottom of the tube, and leaving the lid unheated is acceptable. During a 30‐min incubation at 37°C, almost no condensation is observed on the lid. In contrast, using a standard 37°C incubator is less ideal because the first and second time points occur at 5 and 10 min, respectively, making accurate sample collection difficult when the entire rack must be moved in and out as a whole. Delays longer than 1 min are not acceptable. Shaking or stirring is not required during incubation.
12 Heat all samples at 95°C for 5 min and then centrifuge at ≥16,000 rcf, 25°C, for 3 min; load the supernatant onto an SDS‐PAGE gel; and run at 180 V for 20‐25 min (as described in Basic Protocol 1, step 12).
13 Excise the gel regions above 25 kDa and below 10 kDa to retain only the 10‐25 kDa region for membrane transfer, as described in Basic Protocol 1, step 13.
14 Prepare 5% BSA in 1× TBST, either freshly or stored up to 1 week at 4°C; block the membrane by incubating in 5% BSA in 1× TBST for 1 hr at room temperature; and rinse briefly with 1× TBST (as described in Basic Protocol 1, step 14).
15 Dilute the anti‐PTM primary antibody in 5% BSA in 1× TBST (typically 1:2000‐1:5000) and incubate overnight at 4°C; in parallel, incubate the membranes to be used for loading control with an anti‐histone antibody, i.e., H2B, H3, or H4, depending on the specific histone carrying the modification, as described in Basic Protocol 1, step 15.
  A separate gel should always be run for anti‐histone Western blotting as a loading control. Stripping and re‐probing is not recommended, as it often fails to completely remove the initial antibody signal. In free histone protein assays, the anti‐histone signal generally does not remain stable over time and can vary significantly. This variability depends on the histone subtype (e.g., H3, H4, H2B) as well as the type and position of the PTM. Therefore, each replicate should be run separately for the anti‐histone blot despite concerns about conserving material. Although membranes are not stripped, individual time points must still be normalized to the corresponding anti‐histone signal during data quantification.
16 The following day, wash the membrane with 1× TBST and then incubate with the appropriate secondary antibody at room temperature for 1 hr, as described in Basic Protocol 1, step 16.
17 Apply ECL solution and visualize the signal using an imager as described in Basic Protocol 1, step 17.
18 Collect gel image, quantify bands using ImageJ, and perform calculations using GraphPad Prism, as described in Basic Protocol 1, step 18.

## Basic Protocol 3 *K*_M_ MEASUREMENT FOR DEACYLATION ASSAY ON NUCLEOSOME OR COFACTOR

The key to determining *K*_M_ is to measure the *V*/[E] across varying concentrations of NCP or cofactors such as NAD (for SIRT) (see Strategic Planning, “Nucleosome kinetic theory”). The setup procedure for each specific assay follows the same steps as described in Basic Protocol 1 for the NCP assay, and the materials are as in Basic Protocol 1.

### Additional Materials (also see Basic Protocol 1)

Stock solution of cofactor to be mentioned: e.g., NAD stock solution (see recipe)

1 Just before setting up the assay, determine the concentrations of HDAC complexes or SIRTs in the samples using SDS‐PAGE followed by Coomassie Blue staining, with a gradient of BSA as the standard for quantification, as described in Basic Protocol 1, step 1.
2 Measure the NCP concentrations using a NanoDrop spectrophotometer as described in Basic Protocol 1, step 2.
3 Plan the assay based on the freshly measured concentration.
  Once we obtain the concentrations of HDAC complexes or SIRTs and NCPs, it is important to plan the reaction carefully and calculate all required components. As an example, the measurement of K_M_ for SIRT6 diacylation on H3K9ac‐NCP is presented (the same approach applies to NAD K_M_ determination). The assay setup using varying NCP concentrations—10, 20, 50, 100, 200, and 400 nM—is detailed in Tables 9, 10, 11, 12. Specifically, 2 nM SIRT6 is used with 10 nM and 20 nM NCP substrates (Table 9); 10 nM SIRT6 is used with 50 nM, 100 nM, and 200 nM NCP substrates (Tables 10 and 11); and 40 nM SIRT6 is used with the 400 nM NCP substrate (Table 12). For each reaction, samples will be collected at 0, 30, 60, 90, and 120 min. Therefore, the reaction mix is calculated and prepared in sufficient volume to accommodate sampling at more than five time points.
  For a standard SIRT6 deacylation assay on 100 nM NCP, 5 µl of sample is mixed with 5 µl DQ buffer, and 5 µl of this 1:1 sample/DQ mixture per time point is loaded per lane as mentioned above. However, for K_M_ measurement from a deacylation assay on NCP involving varying final concentrations of NCP, different volumes are loaded onto the SDS‐PAGE gel to also ensure clear Western blot signals and quantifiable trends (see tables for detail). The final loading volume may remain the same through additional DQ dilutions or increase due to a larger total reaction volume when collecting samples at each time point. Consequently, all calculations must be adjusted accordingly prior to assay setup. For example, in SIRT6 deacylation assay on 200 nM NCP (Table 11), 5 µl of sample is mixed with 15 µl of DQ buffer, and 5 µl of this 1:3 sample/DQ mixture per time point is loaded per lane. In SIRT6 deacylation assay on 400 nM NCP (Table 12), 5 µl of sample is mixed with 35 µl of DQ buffer, and 5 µl of this 1:7 sample/DQ mixture per time point is loaded per lane. This ensures clear Western blot signals and maintains measurements within the confirmed linear antibody detection range for accurate quantification (see Strategic Planning, “Pre‐characterization of site‐specific antibodies by Western blotting”).

**Table 9:** Example Setup of a SIRT6 Deacetylation Assay on 10 nM and 20 nM H3K9ac‐NCP in Duplicate

**Reagent**	**Stock**	**Working stock**	**Volume**	
H3K9ac‐NCP	7.28 µM	400 nM	1.21 µl	
1× SIRT reaction buffer			20.79 µl	
Total			22.0 µl	
	**H3K9ac‐NCP 10 nM**		**H3K9ac‐NCP 20 nM**	
**Reagent**	**Rep 1**	**Rep 2**	**Rep 1**	**Rep 2**
1× SIRT reaction buffer	152.8 µl	152.8 µl	111.6 µl	111.6 µl
H3K9ac‐NCP	4.0 µl	4.0 µl	6.0 µl	6.0 µl
100 nM SIRT6	3.2 µl	3.2 µl	2.4 µl	2.4 µl
Total	160 µl	160 µl	120 µl	120 µl

H3K9ac‐NCP is diluted to 400 nM first. *NOTE*: For the 10 nM NCP assay, 30 µl of the reaction sample is mixed with 30 µl of DQ buffer at each time point, and 30 µl of this mixture is loaded onto the gel. For the 20 nM NCP assay, 20 µl of the reaction sample is mixed with 20 µl of DQ buffer, and 20 µl of the mixture is loaded onto the gel.

**Table 10:** Example Setup of a SIRT6 Deacetylation Assay on 50 nM and 100 nM H3K9ac‐NCP in Duplicate

**Reagent**	**Stock**	**Working stock**	**Volume**	
H3K9ac‐NCP	10.5 µM	1.0 µM	6.1 µl	
1× SIRT reaction buffer			57.9 µl	
Total			64.0 µl	
	**H3K9ac‐NCP 50 nM**		**H3K9ac‐NCP 100 nM**	
**Reagent**	**Rep 1**	**Rep 2**	**Rep 1**	**Rep 2**
1× SIRT reaction buffer	34 µl	34 µl	32 µl	32 µl
H3K9ac‐NCP	2.0 µl	2.0 µl	4.0 µl	4.0 µl
100 nM SIRT6	4.0 µl	4.0 µl	4.0 µl	4.0 µl
Total	40 µl	40 µl	40 µl	40 µl

H3K9ac‐NCP is diluted to 1.0 µM first. *NOTE*: For both the 50 nM and 100 nM NCP assays, 5 µl of the reaction sample is mixed with 5 µl of DQ buffer at each time point, and 5 µl of the resulting mixture is loaded onto the gel.

**Table 11:** Example Setup of a SIRT6 Deacetylation Assay on 200 nM H3K9ac‐NCP in Duplicate

**Reagent**	**Stock**	**Working stock**	**Volume**
H3K9ac‐NCP	7.28 µM	1.0 µM	4.7 µl
1× SIRT reaction buffer			29.5 µl
Total			34.2 µl
	**H3K9ac‐NCP 200 nM**		
**Reagent**	**Rep 1**	**Rep 2**	
1× SIRT reaction buffer	30 µl	30 µl	
H3K9ac‐NCP	8.0 µl	8.0 µl	
100 nM SIRT6	2.0 µl	2.0 µl	
Total	40 µl	40 µl	

H3K9ac‐NCP is diluted to 1.0 µM first. *NOTE*: For the 200 nM NCP assay, 5 µl of the reaction sample is mixed with 15 µl of DQ buffer at each time point, and 5 µl of the resulting mixture is loaded onto the gel.

**Table 12:** Example Setup of a SIRT6 Deacetylation Assay on 400 nM H3K9ac‐NCP in Duplicate

**Reagent**	**Stock**	**Working stock**	**Volume**
H3K9ac‐NCP	7.28 µM	7.28 µM	No dilution
Total			10.2 µl
	**H3K9ac‐NCP 400 nM**		
**Reagent**	**Rep1**	**Rep2**	
1× SIRT reaction buffer	29.8 µl	29.8 µl	
H3K9ac‐NCP	2.2 µl	2.2 µl	
100 nM SIRT6	8.0 µl	8.0 µl	
Total	40 µl	40 µl	

H3K9ac‐NCP is not diluted as the original 7.28 µM stock solution is used here. *NOTE*: For the 400 nM NCP assay, 5 µl of the reaction sample is mixed with 35 µl of DQ buffer at each time point, and 5 µl of the resulting mixture is loaded onto the gel.

4 Freshly prepare 1 ml total of the appropriate 1× reaction buffer—1× HDAC reaction buffer for HDAC complexes or 1× SIRT reaction buffer for SIRTs—for all subsequent dilutions as described in Basic Protocol 1, step 4. Prepare 1× reaction buffer containing varying concentrations of the cofactor to be measured, such as NAD.
5 Continue with steps 5‐12 of Basic Protocol 1.
6 Excise the gel regions above 25 kDa and below 10 kDa to retain only the 10‐25 kDa region for membrane transfer, as described in Basic Protocol 1, step 13.
  As outlined in Basic Protocol 1, no more than 12 assays should be set up concurrently, as the assay is time sensitive. K_M_ measurement is also batch sensitive, try to use the same 1× reaction buffer and same batch of NCP as well as enzyme if possible. If it is necessary to perform more than 12 assays, the 1 × reaction buffer may be stored at 4°C overnight and remains suitable for use the following day.
  When comparing different NCP concentrations or NAD concentrations with the same enzyme, it is advisable to transfer multiple gels onto a single membrane for direct comparison. Use distinct molecular‐weight‐marker patterns to distinguish each gel after transfer. Clear and consistent labeling, especially for the t = 0 time point, is critical for accurate interpretation. Marker patterns (e.g., lane position, number of bands, band intensities) can be used to differentiate gels or indicate orientation.
7 Continue with steps 14‐19 of Basic Protocol 1.

## Basic Protocol 4 ASSESSMENT OF DEACYLATION INHIBITOR OR ACTIVATOR EFFECTS ON NCP SUBSTRATES

An increasing body of evidence suggests that drug development targeting various HDAC complexes or SIRTs should be based on NCP assays rather than histone peptides or free histone proteins (Wang et al., 2025; Abeywardana et al., 2024). This is due to the complex nature of interactions between enzymes and NCPs, which involve both histones and DNA and more accurately recapitulate the cellular chromatin environment. In our own studies, several small‐molecule regulators have shown dramatic differences in IC_50_ or EC_50_ values, with some even exhibiting completely opposite effects. For example, CL‐5D functions as an activator in histone peptide assays but as an inhibitor in NCP‐based assays (Wang et al., 2025).

To accurately determine IC_50_ or EC_50_ values, the *V*/[E] should be measured across a range of inhibitor or activator concentrations (see Strategic Planning, “Nucleosome kinetic theory”). The assay setup generally follows the same procedures as described in Basic Protocol 1 for NCP‐based assays.

This protocol can also be applied to free histone protein assays. However, due to the inherent challenges of histone protein assays (described in Basic Protocol 2), obtaining a clear trend in a series of deacylation assays on free histone proteins is even more difficult. Therefore, such experiments are not recommended unless necessary (Wang et al., 2025).

### Materials (also see Basic Protocol 1)

Inhibitors and activators such as MS‐275 (HDAC inhibitor), IRBM6 (HDAC inhibitor; Shiota et al., 2021), MDL‐800 (SIRT6 activator), and CL‐5D (SIRT6 inhibitor); MDL‐800 (Sigma cat. no. SML2529‐5MG) is used as an example here
Dimethyl sulfoxide (DMSO; Sigma, cat. no. D4540)

1 Just before setting up the assay, determine the concentrations of HDAC complexes or SIRTs in the samples using SDS‐PAGE followed by Coomassie Blue staining, with a gradient of BSA as the standard for quantification, as described in Basic Protocol 1, step 1.
2 Measure NCP concentrations using a NanoDrop Spectrophotometer as described in Basic Protocol 1, step 2.
3 Plan the assay based on the freshly measured concentration.
  To observe a clear trend when measuring IC_50_ or EC_50_, it is essential to first determine the K_M_ of NCP (see Basic Protocol 3). This is because using [NCP] near its K_M_, rather than at saturating levels, allows sufficient dynamic range in V/[E] for detecting both inhibition and activation. A preliminary assay without inhibitor or activator should be conducted to identify the optimal enzyme concentration that yields clear Western blot signals and quantifiable trends. For example, when evaluating MDL‐800 in a SIRT6 assay, we first determined the K_M_ for H3K9ac‐NCP to be ∼20 nM and then optimized assay conditions using 2 nM SIRT6 with 20 nM NCP.
  To generate a reliable IC_50_ or EC_50_ curve, a logistic (sigmoidal) dose‐response is required. Therefore, a serial dilution of the inhibitor or activator must be prepared in advance. DMSO should be used as the vehicle control, and all assay conditions should maintain the same final DMSO concentration across all inhibitor or activator groups.
4 Prepare appropriate 1 ml total of 1× reaction buffer (1× HDAC reaction buffer for HDAC complexes or 1× SIRT reaction buffer for SIRTs) as described in Basic Protocol 1, step 4.
5 Prepare and aliquot DQ solution according to the assay design as described in Basic Protocol 1, step 5.
6 Prepare a 1 µM working stock solution of NCP in 1× reaction buffer, mix thoroughly, centrifuge briefly, and keep on ice until use, as described in Basic Protocol 1, step 6.
7 Dilute the HDAC complexes or SIRTs to the desired concentration according to the experimental design as working stock solution in 1 × Reaction Buffer. Mix thoroughly, centrifuge briefly, and keep the solution on ice until use, as in Basic Protocol 1, step 7.
8 Dissolve the inhibitor or activator to be tested (for example, MDL‐800) in DMSO to prepare a stock solution. Prepare a serial dilution in DMSO to obtain the working concentrations (Table 13). Add the diluted solutions to reaction tubes 1‐7 containing 1× reaction buffer according to the experimental design from step 2. Mix thoroughly, centrifuge briefly, and keep the solution on ice until use. Use buffer containing 10% DMSO as a vehicle control, and buffer without DMSO as a positive control.
  The preparation method depends on the aqueous solubility of the inhibitor or activator. Ideally, a lower DMSO percentage minimizes its effect on enzymes. If the compound is fully water soluble, prepare both the stock solution and serial dilutions in 1× reaction buffer. If it is less soluble, prepare the stock in DMSO but perform all serial dilutions in 1× reaction buffer. However, because solubility can be difficult to judge at small volumes, to ensure complete dissolution, prepare and dilute entirely in DMSO, keeping the final DMSO concentration consistent across all samples.
  DMSO in the assay may affect enzyme activity. Unless the effect is significant, use same amount of pure DMSO with no inhibitor or activator diluted into the reaction solution as the control, calculate its V/[E] value, and use it to normalize the results for further analysis and curve fitting. If the enzyme is strongly inhibited or activated by DMSO, refer to the Troubleshooting section.

**Table 13:** Example Setup of an MDL‐800‐inhibited SIRT6 Deacetylation Assay on 20 nM H3K9ac‐NCP

**Stock**	Stock 1	Stock 2	Stock 3	Stock 4	Stock 5	Stock 6	
Preparation	Dissolve MDL‐800 in DMSO	Add 10 µl stock 1 to 90 µl DMSO	Add 10 µl stock 2 to 90 µl DMSO	Add 10 µl stock 3 to 90 µl DMSO	Add 10 µl stock 4 to 90 µl DMSO	Add 10 µl stock 5 to 90 µl DMSO	
Concentration	10 mM	1 mM	100 µM	10 µM	1 µM	0.1 µM	
**Tube**	1	2	3	4	5	6	7
**MDL‐800 final concentration**	DMSO	100 µM	10 µM	1 µM	0.1 µM	0.01 µM	No DMSO
1× SIRT reaction buffer	70 µl	70 µl	70 µl	70 µl	70 µl	70 µl	79 µl
DMSO	9.0 µl	0 µl	0 µl	0 µl	0 µl	0 µl	0 µl
MDL‐800	0 µl	9.0 µl stock 2	9.0 µl stock 3	9.0 µl stock 4	9.0 µl stock 5	9.0 µl stock 6	0 µl
90 nM SIRT6	2.0 µl	2.0 µl	2.0 µl	2.0 µl	2.0 µl	2.0 µl	2.0 µl
200 nM NCP	9.0 µl	9.0 µl	9.0 µl	9.0 µl	9.0 µl	9.0 µl	9.0 µl
Total	90 µl	90 µl	90 µl	90 µl	90 µl	90 µl	90 µl

MDL‐800 is first dissolved in DMSO to prepare a 10 mM stock solution. This stock is then serially diluted in DMSO to generate working solutions at final concentrations of 1 mM, 100 µM, 10 µM, 1 µM, and 0.1 µM. *NOTE*: For this deacylation assay, we used 2 nM SIRT6 and different concentrations of MDL‐800 ranging from 0.1 µM to 100 µM, at each time point, 20 µl sample is mixed with 20 µl DQ to load 20 µl on gel.

9 Dilute the HDAC complexes or SIRTs to the desired final concentrations into each tube 1‐7 according to the experimental design from **s**tep 2. Mix thoroughly, centrifuge briefly, and keep the solution on ice until use.
10 Allow tubes 1‐7 to warm to room temperature and pre‐incubate for 5 min to facilitate slow binding. Cool the tubes on ice for 3 min and then add NCP to a final concentration of 20 nM to initiate the deacylation reaction. Mix thoroughly, centrifuge briefly, and keep the solution on ice until use.
11 Take the *t* = 0 sample by mixing 1:1 with DQ buffer, heat at 95°C for ∼1 min to quench, and store the sample on ice, as described in Basic Protocol 1, step 11. Incubate the reaction at 37°C and collect samples at 30, 60, 90, and 120 min. Quench each time point by mixing 1:1 with DQ buffer, briefly boiling for ∼1 min, and then storing on ice.
  The time interval may vary depending on the effects of different inhibitors or activators. To run three assays on a single gel, samples are typically taken at 0, 30, 60, and 120 min, with the 90‐min time point omitted when necessary.
12 Continue with Basic Protocol 1, steps 12‐19.
  For a complete inhibitor or activator analysis on NCP, two biological replicates are generally required, as in Basic Protocol 1, because of the difficulty of working with site‐specific PTM‐modified NCP. To ensure reproducibility and scientific rigor, the center of the S‐shaped curve should include at least one data point near the IC50 or EC50, plus one data point above and one below to capture the trend. The head and tail regions of the curve should each contain at least one data point; in these plateau regions, a single replicate may be sufficient if the results are clearly consistent.

## REAGENTS AND SOLUTIONS

### DQ buffer

2× Laemmli buffer
20 mM EDTA (Sigma, cat. no. E9884)
  Laemmli buffer may be diluted from 4× Laemmli buffer (Bio‐Rad, cat. no. 1610747) or from homemade 4× SDS loading buffer.

### HDAC reaction buffer, 1×, pH ∼7.5, ∼–69 mV

50 mM HEPES, pH 7.5 (Thermo Scientific, cat. no. J60712.AP)
100 mM KCl (Thermo Scientific, cat. no. A11662.0B)
100 µM IP6 (Sigma, cat. no. P8810)
0.2 mg/ml BSA (GeminiBio, cat. no. 700‐100P; prepare 2 mg/ml stock and store at –80°C)
  When preparing the reaction buffer, always add water first, followed by other components in order from largest to smallest volume.
  HEPES buffer may be stored at room temperature but must be confirmed to be at pH 7.5 (ion strength = ∼‐69 mV) for each batch of final 1× reaction buffer after setup.

### IP6 stock solutions, 20 mM and 2 mM

Commercial IP6 from Sigma‐Aldrich (cat. no. P8810) is phytic acid sodium salt hydrate, with the formula C_6_H_18_O_24_P_6_·*x*Na^+^·*y*H_2_O and a molecular weight of 660.04 Da (anhydrous free acid basis). Because it is a mixture with variable *x* and *y* values, the exact molecular weight cannot be determined. However, because it is used here solely as a stabilizing agent for the CoREST complex structure, exact accuracy is not essential. For simplicity, we assume *x* = 1 and *y* = 1, giving an adjusted molecular weight of 700.04 Da. To prepare a 20 mM solution, dissolve 14.0 mg in 1.0 ml of water, aliquot into ten portions to achieve a final concentration of 2.0 mM per aliquot, and store at –80°C. Alternatively, purchasing from a different source can resolve this issue, for example C_6_H_12_O_24_P_6_•6Na from Cayman Chemical (cat. no. 10008415), which has a defined molecular weight of 791.9 Da.

### NAD stock solution, 50 mM

To prepare a 50 mM NAD stock solution, dissolve 34.3 mg of NAD (Sigma, cat. no. NAD100‐RO) in 1 ml of water. Dilute this stock solution 100‐fold to obtain a working solution. Measure the absorbance of the diluted solution at 260 nm using a NanoDrop spectrophotometer, recording *A*_260_ four times. Calculate the average and determine the concentration using the Lambert‐Beer law: [NAD] = *A*_ave_/(ε·*b*), where the pathlength (*b*) is 1 cm and the molar extinction coefficient ε for NAD is 16,900 L·mol^−1^ cm^−1^.

### SIRT reaction buffer, 1×, pH ∼7.5, ∼–69 mV

50 mM HEPES, pH 7.5
1 mM NAD
1 mM dithiothreitol (DTT; GoldBio, cat. no. DTT10)
0.2 mg/ml BSA
  When preparing the reaction buffer, always add water first, followed by other components in order from largest to smallest volume.
  HEPES buffer may be stored at room temperature but must be confirmed to be at pH 7.5 (ion strength = ∼‐69 mV) for each batch of final 1× reaction buffer after setup. A 10 mM DTT stock solution may also be prepared, aliquoted, and stored up to 1 month at –80°C.

### SDS running buffer, 10×

30.3 g Tris base (Sigma, cat. no. T1503)
150.1 g glycine (VWR, cat no. 0167‐1KG)
10 g SDS (Amresco, cat. no. M107)
Dissolve in ∼900 ml Milli‐Q water to 1 L final

### TBE buffer, 10×

108 g Tris base (Sigma, cat. no. T1503)
55 g boric acid (Fisher, cat. no. A73‐500)
20 ml EDTA (Sigma, cat. no. E9884)
Dissolve in ∼480 ml Milli‐Q water to 500 ml final
Adjust to pH 8.0 using concentrated HCl

### TBST running buffer, 10×

24 g Tris base (Sigma, cat. no. T1503)
88 g NaCl (VWR, cat. no. BDH9286)
10 ml Tween‐20 (Sigma, cat. no. P1379)
Dissolve in ∼900 ml Milli‐Q water to 1 L final
Adjust to pH 7.4 using concentrated HCl

## COMMENTARY

### Background Information

The nucleosome‐based deacylation assays described here provide a mechanistically informative platform that bridges traditional biochemical assays and chromatin‐level enzymology. Unlike with traditional peptide or free‐histone systems, the use of chemically defined NCPs enables direct assessment of enzyme‐chromatin interactions under conditions that mimic physiological substrate organization. This consideration has become increasingly important, particularly when investigating large chromatin‐binding complexes involved in transcriptional regulation, DNA replication, or repair. Only modern NCP‐based enzymology can truly match the advances achieved in structural studies such as cryo‐EM, allowing mechanistic understanding at both biochemical and structural levels in the post‐genomic era.

These four protocols enable quantitative determination of kinetic parameters such as *k*_cat_ and *K*_M_, offering mechanistic insight into how HDAC complexes and SIRTs engage with chromatin‐like substrates and how cofactors, small‐molecule regulators, or binding partners modulate catalysis. Comparison of HDAC or SIRT activity on NCPs versus free histones reveals allosteric and surface‐binding effects contributed by the histone octamer and DNA, which are not captured in peptide‐based assays. Although demonstrated here for deacetylation by HDAC complexes and SIRTs, this experimental framework can be readily adapted to study other PTMs using any “eraser” or “writer” enzymatic assays.

Mechanistically, the kinetic framework described here complements other biophysical methods such as electrophoretic mobility shift assays (EMSA) and structural studies (e.g., cryo‐EM or X‐ray crystallography). Whereas structural approaches capture static enzyme‐substrate complexes, the kinetic assays presented here quantify their dynamic behavior, providing rate‐based insights into substrate turnover, complex stability, and active‐site accessibility in a systematic and integrative manner. Although less precise than structural analyses and more experimentally challenging than traditional enzymology, NCP‐based kinetics uniquely enables the investigation of chromatin‐associated enzymatic reactions under chemically defined, cell‐free conditions. This approach provides otherwise inaccessible macromolecular insights, facilitating comparison of enzyme families and revealing PTM trends within a biologically relevant, yet mechanistically interpretable framework.

From a pharmacological perspective, these protocols provide a systematic framework for evaluating inhibitor and activator potency under chromatin‐relevant conditions. Because many small molecules exhibit distinct behaviors on nucleosome substrates compared to peptide assays, this workflow establishes a more physiologically meaningful benchmark for screening and mechanistic validation. Beyond HDACs and SIRTs, the same strategy can be broadly applied to other chromatin‐modifying enzymes, thereby facilitating drug discovery and advancing structure‐guided design of chromatin‐targeting therapeutics.

### Troubleshooting

Tables 14, 15, 16, and 17 lists problems that may arise with Basic Protocols 1, 2, 3, and 4, respectively, along with their possible causes and solutions.

**Table 14:** Troubleshooting for Basic Protocol 1

Step	Problem	Possible cause	Solution
1	The band intensities of different components within the HDAC complex are not present in a 1:1:1 ratio.	This occurs because HDAC complexes expressed in mammalian systems are often not strictly stoichiometric and/or Coomassie blue staining can be somewhat variable based on protein sequence.	Quantification is performed using only the HDAC1 band.
11	Some solution is visible on the tube wall.	Excessive disturbance during sampling or the tube was accidentally dropped after closing.	A brief centrifugation is usually sufficient to bring the solution back to the bottom of the tube.
17	The two replicates show inconsistent results.	Gel loading error or uneven membrane transfer.	Pay close attention to pipetting technique, as emphasized in the detailed procedure above. Repeat the entire assay to verify results.
17	Band intensity remains unchanged or even increases instead of decreasing.	The enzyme concentration is too low, resulting in a slow reaction rate.	Increase the enzyme concentration and repeat the entire assay. In general, we do not exceed a final concentration of 120 nM in order to observe at least one turnover.
18	One band appears significantly weaker, stronger, or has a bubble.	Gel loading inconsistency or uneven membrane transfer.	(1) If the affected band corresponds to the *t* = 0 time point, the assay should be repeated. (2) If it is not the *t* = 0 band and the deviation is not substantial, the result can be accepted as part of random experimental error. However, if the intensity deviates by more than 100%, rerun the same remaining samples for Western blotting to verify.
18	Anti‐PTM band intensity appears unevenly distributed (some too intense or too faint).	Inaccurate pipetting or uneven membrane transfer.	Carefully standardize pipetting technique; if variability exceeds acceptable range (e.g., >2‐fold), consider reloading or re‐running the affected samples.
18	Reaction rate does not scale linearly with enzyme concentration.	Pipetting error or uneven membrane transfer; alternatively, enzyme activity may be inherently nonlinear at tested concentrations.	(1) Double‐check pipetting accuracy and membrane transfer consistency (2) If the issue persists after repeating, test a broader range of enzyme concentrations (3) Nonlinear behavior may reflect intrinsic enzyme kinetics under current assay conditions
18	Anti‐histone blot signal increases or decreases significantly.	Uncommon in NCP assays; may result from pipetting errors or uneven membrane transfer.	Carefully review pipetting technique and membrane handling; rerun the same remaining samples for Western blotting to verify.
18	After 2 hr of assay, anti‐PTM band intensity does not approach zero but plateaus at a certain level.	It is acceptable for bands not to reach zero for kinetic analysis; however, a complete plateau suggests potential substrate inhibition or loss of enzyme activity due to instability. This is rare in NCP assays, which generally show good stability.	(1) If the plateau occurs at a high intensity (>50% of the *t* = 0 band), increase the enzyme concentration. (2) If the plateau occurs at a low intensity (<30% of the *t* = 0 band), lower the enzyme concentration. (3) If plateaus persist, shorten the reaction time.
19	Native gel shows a strong free DNA band.	Some free DNA is expected, as NCP can partially dissociate during native PAGE. However, if the free DNA band is overly intense (e.g., >50%), even in the positive control, this suggests a problem.	Evaluate NCP integrity using an additional gel. If poor quality persists, remake the NCP.

**Table 15:** Troubleshooting for Basic Protocol 2, *^a^*

Step	Problem	Possible cause	Solution
2	Free histone protein does not dissolve well in water, and SDS‐PAGE shows a weak band.	This often occurs with redissolved histone proteins that were previously lyophilized from old stock solutions.	Dissolve histone in 6‐7 M guanidine HCl to fully unfold, then dialyze into 0.1% trifluoroacetic acid (TFA) and lyophilize to obtain a dry powder. Prepare a fresh stock solution from this powder, and centrifuge before use to remove any precipitate that may interfere with enzymatic assays.
11	Small droplets of solution accumulate on the tube lid when using a heating block.	This is common with heating blocks because the lids are not heated. It may also result from excessive stirring or shaking during sample handling.	(1) If the droplet is small, it can be ignored. (2) If multiple droplets form, briefly centrifuge the tube to collect the contents. (3) If this problem occurs frequently, consider switching from a heating block to an incubator to avoid condensation on the lid.
17	Anti‐PTM band intensity is very low.	This issue is not commonly observed with NCPs or when using certain widely validated antibodies (such as anti‐K9ac or anti‐K27ac). It may result from differences in folding states between NCPs and free histone proteins, or from precipitation of free histones.	(1) Check the anti‐H3 band (see next question). If necessary, increase exposure time to boost signal intensity. (2) If the signal remains weak, extend transfer time. Use a stronger ECL detection reagent (as observed in the H2BK46ac protein assay; Wang et al., 2022). (3) If the problem persists, try an alternative antibody from another supplier.
17	Anti‐histone band intensity decreases dramatically.	As noted above, the exact reason is unclear, but free histone proteins tend to precipitate more readily than NCPs.	Shorten the reaction time to 0, 2, 4, 8, and 20 min (as demonstrated in the H2BK11ac protein assay; Wang et al., 2022).
17	Inconsistent results between two replicates	More common in histone protein assays than in NCP assays, likely due to uneven gel loading, inconsistent membrane transfer, or slight variations in sample handling.	Pay close attention to pipetting accuracy. Rerun the same remaining samples for Western blotting to verify. If results remain inconsistent, repeat the entire assay. Ensure that samples are taken at consistent time intervals (e.g., every 15 s).
17	Anti‐PTM band intensity remains constant or increases instead of decreasing.	More common in histone protein assays than in NCP assays; likely due to insufficient enzyme concentration resulting in a low reaction rate.	Increase the enzyme concentration and repeat the entire assay.
17	Still no significant decrease in anti‐PTM band intensity after increasing enzyme concentration.	This issue occurs more often in histone protein assays than in NCP assays, likely due to the inherently slow activity of HDAC complexes on free histone proteins. Additionally, the histone substrate concentration in free histone assays is typically 1000 nM compared to 100 nM in NCP assays, making small degrees of deacylation less apparent.	(1) Continue increasing enzyme concentration and repeat the entire assay but generally do not exceed a final concentration of 300 nM, which should be sufficient to observe at least one turnover. (2) If no change is observed at 300 nM, extend the incubation time to up to 2 hr if the free histone protein is stable. (3) If the protein is unstable or no change is still observed, report no significant activity.
18	One band appears significantly weaker, stronger, or shows a bubble.	This is more frequently observed in histone protein assays than in NCP assays, often due to uneven gel loading or membrane transfer.	(1) If the affected band corresponds to the *t* = 0 time point, the assay must be repeated. (2) If it is not the *t* = 0 band and the deviation is minor, it can be accepted as part of random error. (3) If the deviation is >100%, rerun the same remaining samples for Western blotting to verify.
18	Anti‐PTM band intensity fluctuates unevenly.	This occurs more often in histone protein assays than in NCP assays, typically due to pipetting inaccuracies or uneven membrane transfer.	(1) Ensure precise pipetting; practice and repeat the entire assay if necessary. (2) If fluctuations persist, try increasing enzyme concentration. (3) If no clear trend emerges after adjustments, report as no significant activity.
18	The reaction rate does not scale linearly with enzyme concentration.	Due to the inherent challenges of histone protein assays, a linear relationship test is generally unnecessary, except in specific cases (e.g., MIER protein assay on H3K9ac; see Wang et al., 2022). Potential causes include pipetting errors or uneven membrane transfer.	Ensure precise pipetting and maintain consistent handling techniques. If the issue persists after repeating the assay, test both higher and lower enzyme concentrations. In some cases, non‐linear activity may result from differences in substrate binding between free histone proteins and NCPs.
18	After 2 hr of assay, anti‐PTM band intensity does not approach zero but plateaus at a certain level.	It is acceptable for bands not to reach zero for kinetic analysis. However, a complete plateau suggests potential substrate inhibition or loss of enzyme activity due to instability. This behavior is more common with free histone protein assays than with NCP assays.	(1) If the plateau is high (>50% of the *t* = 0 band): increase the enzyme concentration. If a plateau persists, shorten the reaction time. (2) If the plateau is low (<30% of the *t* = 0 band), reduce the enzyme concentration. If it still plateaus, proceed with analysis as is.

^*a*^ Some common questions covered in Table 14 are not repeated here.

**Table 16:** Troubleshooting for Basic Protocol 3, *^a^*

Step	Problem	Possible cause	Solution
17	Anti‐PTM band intensity increases or stays constant instead of decreasing.	Enzyme concentration is too low, especially when NCP concentration is unusually high, making the fraction of deacylated substrate small.	Increase the enzyme concentration and repeat the entire assay.
18	Anti‐PTM bands appear uneven in intensity or are too faint to detect.	Common at low NCP concentrations due to the need for large loading volumes; may also result from pipetting errors or uneven membrane transfer.	Pay close attention to pipetting technique, as emphasized in the detailed procedure above, and practice for consistency. Increase the loading volume if needed.
18	The anti‐histone bands appear to increase or decrease in intensity.	Common at low NCP concentrations due to the need for large loading volumes; may also result from pipetting errors or uneven membrane transfer.	Pay close attention to pipetting technique, as emphasized in the detailed procedure above. Rerun the same remaining samples for Western blotting to verify.
18	One assay's *V*/[E] value is significantly higher or lower than the overall trend in the series for a Michaelis‐Menten curve analysis.	(1) If the other replicate looks normal, likely due to gel loading inconsistency or uneven membrane transfer; rerun the same remaining samples for Western blotting to verify. (2) If the other replicate is also abnormal, the assay may have been poorly executed, often due to the inherent difficulty at very high or low NCP concentrations.	(1) If the affected point is in the plateau region of the Michaelis‐Menten curve (non‐critical), omit the data. (2) If it is in the early rising phase of the curve, repeat the entire assay.
19	The native gel displays bands that are either too strong or too weak, or it is difficult to distinguish between free DNA, nucleosomes, hexasomes, and sample aggregates.	This is common, because of variation in NCP concentration.	Dilute samples with excessively high concentrations before loading and increase loading volume for samples with low concentrations; however, avoid overloading, as large volumes often produce ‘U’‐shaped bands in native gels.

^*a*^ Step numbers refer to steps of Basic Protocol 1. Some common questions covered in Table 14 are not repeated here.

**Table 17:** Troubleshooting for Basic Protocol 4, *^a^*

Step	Problem	Possible cause	Solution
17	After adding the inhibitor, bands no longer decrease in any assay.	Confirm that the positive control (no inhibitor, no DMSO) shows normal band decrease. If it does, the inhibitor may be too potent or the DMSO concentration too high.	(1) Dilute the inhibitor further to achieve lower final concentrations. (2) If this does not work, reduce the DMSO concentration. Strategy: If the inhibitor is readily available, test its solubility in water. Dissolve it at the highest possible concentration in DMSO, then dilute into a sufficiently large volume of aqueous solution.
17	After addition of the activator, only the *t* = 0 bands are visible in all assays.	Confirm that the positive control (no activator, no DMSO) shows normal band decrease. If it does, the activator may be too powerful at the concentrations selected.	Dilute the activator to achieve lower final concentrations. *NOTE*: DMSO is unlikely to enhance enzyme activity.
18	One assay's *V*/[E] value is significantly higher or lower than the overall trend of the rest in the series.	(1) If the other replicate looks normal, likely due to gel loading inconsistency or uneven membrane transfer; rerun the same remaining samples for Western blotting to verify. (2) If the other replicate is also abnormal, the assay may have been poorly executed, possibly due to serial dilution errors.	(1) If the affected assay is at the head or tail of the S‐shaped curve (non‐critical region), the data can be omitted. (2) If it is in the center of the S‐shaped curve, repeat the entire assay.
18	After calculating the *V*/[E] of all assays, no clear increasing or decreasing trend is observed.	(1) Commonly due to technical errors such as pipetting errors or uneven membrane transfer. (2) Alternatively, the inhibitor or activator is too weak, resulting in only random fluctuations.	(1) Repeat the entire assay to ensure it is not a technical error. (2) For an inhibitor, continue increasing the concentration and repeat the entire assay until the enzyme is fully inhibited. (3) For an activator, continue increasing the concentration and repeat the entire assay until the enzyme activity is increased by at least three‐ to five‐fold to allow EC50 calculation.
18	Anti‐histone Western blot results are consistent within a single assay but vary between assays.	This variation is common in NCP inhibitor or activator assays and may be due to pipetting errors, gel loading inconsistencies, or issues during membrane transfer.	(1) If the variation is minor, accept the results as they are. The overall trend remains unaffected because *V*/[E] is calculated separately for each assay. (2) If the variation is large (e.g., >100%), rerun the same remaining samples for Western blotting to verify or repeat the entire assay. (3) If significant variation persists after repeating, this suggests that the inhibitor or activator may be affecting NCP or histone stability. Refer to the next troubleshooting entry.
19	Native gel shows a strong free DNA band.	Some free DNA is expected, as NCP can partially dissociate during native PAGE. However, if the free DNA band is overly intense (e.g., >50%) even in the positive control, this suggests a problem.	Evaluate NCP integrity using an additional gel. If poor quality persists, remake the NCP. If the NCP control runs well, this suggests that the inhibitor or activator interferes with NCP stability, making the NCP assay unsuitable for assessing this inhibitor or activator.

^*a*^ Step numbers refer to steps of Basic Protocol 1. Some common questions covered in Table 14 are not repeated here.

### Understanding Results and Statistical Analysis

#### Basic Protocol 1

**Figure 3.**
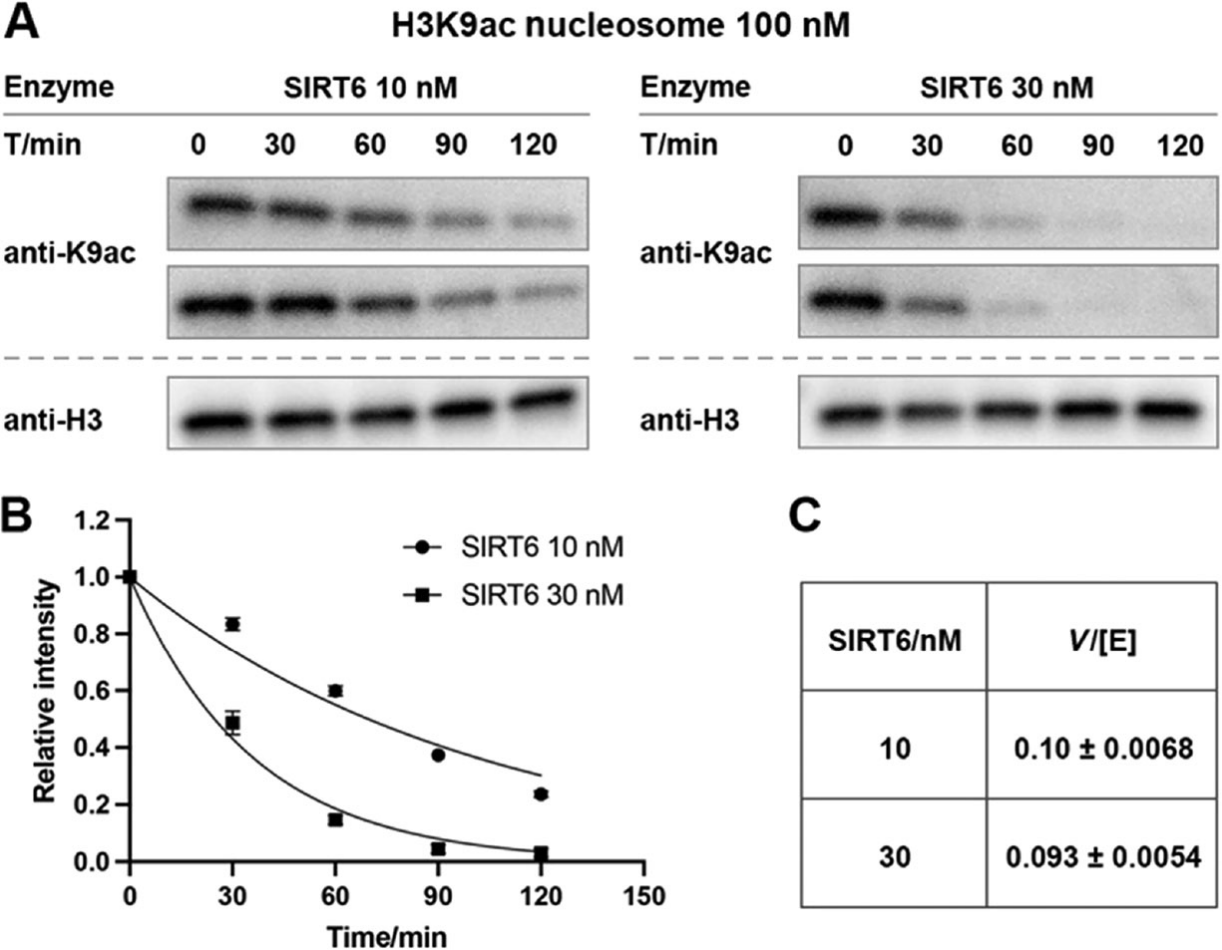
Kinetic analysis of SIRT6 deacetylation of H3K9ac NCP. (**A**) Representative Western blots showing time‐dependent deacetylation of H3K9ac NCP (100 nM) by 10 nM (left) or 30 nM (right) SIRT6. Reactions were sampled at the indicated time points for Western blotting with anti‐H3K9ac antibody. Anti‐H3 was used as a loading control showing no change. (**B**) Quantification of H3K9ac signal normalized to time 0 using ImageJ and fit to a first‐order decay model. Data points represent mean ± SEM from 2 replicates. (**C**) Calculated *V*/[E] ± SEM values for each enzyme concentration. Similar *V*/[E] values at different enzyme concentrations indicate that the reactions were conducted within the linear range.

Quantify histone acetylation band intensity using ImageJ, normalize to the time 0 signal, and fit to a first‐order decay curve to obtain the kinetic parameter *V*/[E] ± SEM value for each enzyme condition (*Y*_0_ constant equal to 1, plateau constant equal to 0, *K* > 0, symmetrical approximate confidence intervals). For the same enzyme tested at different concentrations, if the data fall within the linear range, the *V*/[E] values should be similar. Choose the most reliable dataset as the representative value for reporting (Fig. 3; Wang et al., 2023b).

#### Basic Protocol 2

**Figure 4.**
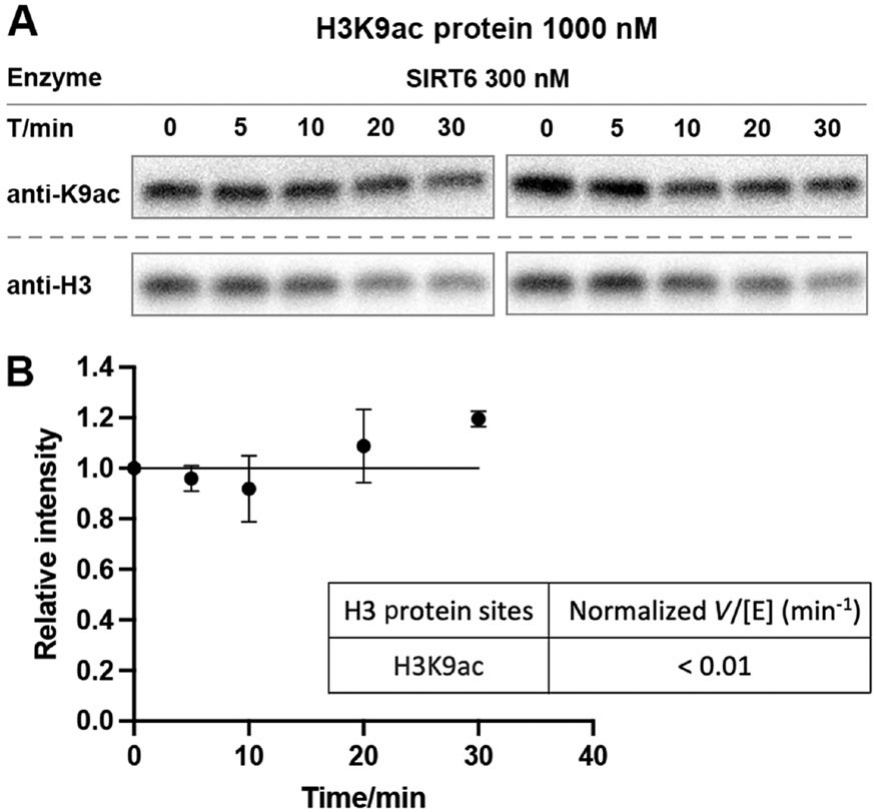
SIRT6 deacetylation of H3K9ac on free histone H3 protein. (**A**) Recombinant H3K9ac protein (1 µM) was incubated with SIRT6 (300 nM) at 37°C for the indicated times. Reactions were sampled at the indicated time points for Western blotting with anti‐H3K9ac antibody. Anti‐H3 was used as a loading control and showed a slight decrease across time points. (**B**) H3K9ac band intensity was quantified using ImageJ, normalized first to the corresponding anti‐H3 band and then to the time 0 signal. Data were fit to a first‐order decay model to determine the kinetic parameter *V*/[E]. The measured normalized *V*/[E] for H3K9ac was <0.01 min^−1^, indicating minimal SIRT6 activity on this substrate. Data points represent *V*/[E] ± SEM of replicate measurements.

Quantify histone acetylation band intensity using ImageJ by first normalizing to the corresponding anti‐histone band, then to the time 0 normalized signal. Fit the resulting values to a first‐order decay curve to determine the kinetic parameter *V*/[E] ± SEM for each enzyme condition (*Y*_0_ constant equal to 1, plateau constant equal to 0, *K* > 0, symmetrical approximate confidence intervals; Fig. 4; Wang et al., 2023b).

#### Basic Protocol 3

**Figure 5:**
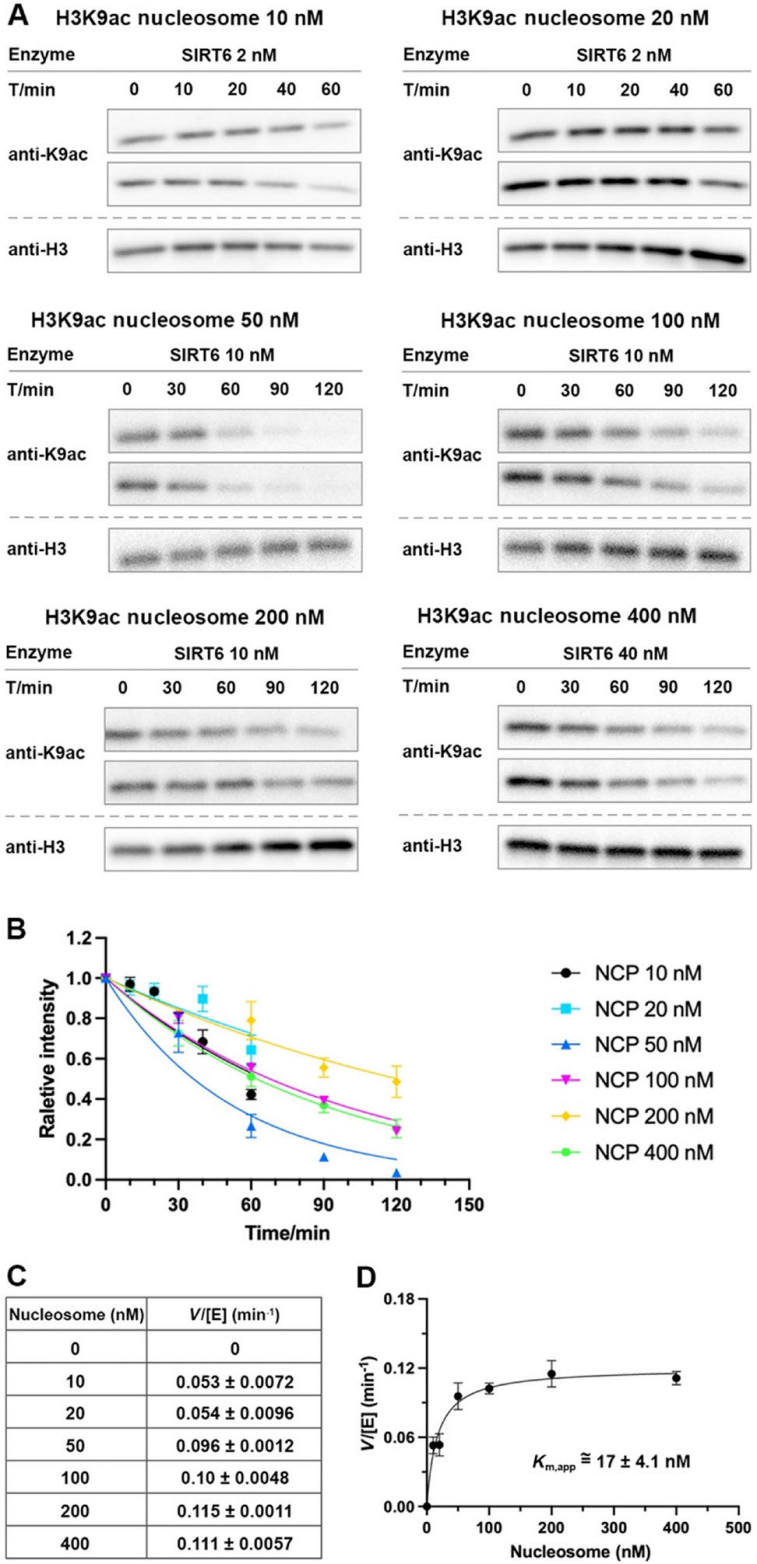
*K*_M_ of SIRT6 for H3K9ac NCP. (**A**) Time‐course Western blot analysis of SIRT6‐mediated deacetylation of H3K9ac NCP at the indicated substrate and enzyme concentrations. Reactions were sampled at the indicated time points for Western blotting with anti‐H3K9ac antibody. Anti‐H3 was used as a loading control and showed no change across time points. (**B**) Quantification of H3K9ac signal intensity was normalized to the time 0 value and fitted to a first‐order decay equation to calculate each *V*/[E] ± SEM. (**C**) Summary of calculated *V*/[E] values for each substrate concentration. (**D**) Michaelis‐Menten plot of *V*/[E] versus NCP concentration, yielding an apparent substrate binding constant (*K*_M,app_) of 17 ± 4.1 nM.

Quantify histone acetylation band intensity using ImageJ, normalize to the time 0 signal, and fit the data to a first‐order decay curve to obtain the *V*/[E] value for each assay. The kinetic parameter *V*/[E] ± SEM for each nucleosome concentration will be then fitted to the Michaelis‐Menten equation to determine *K*_M(NCP)_ and (*V*/[E])_max(NCP)_ (Fig. 5; Wang et al., 2023b).

#### Basic Protocol 4

**Figure 6:**
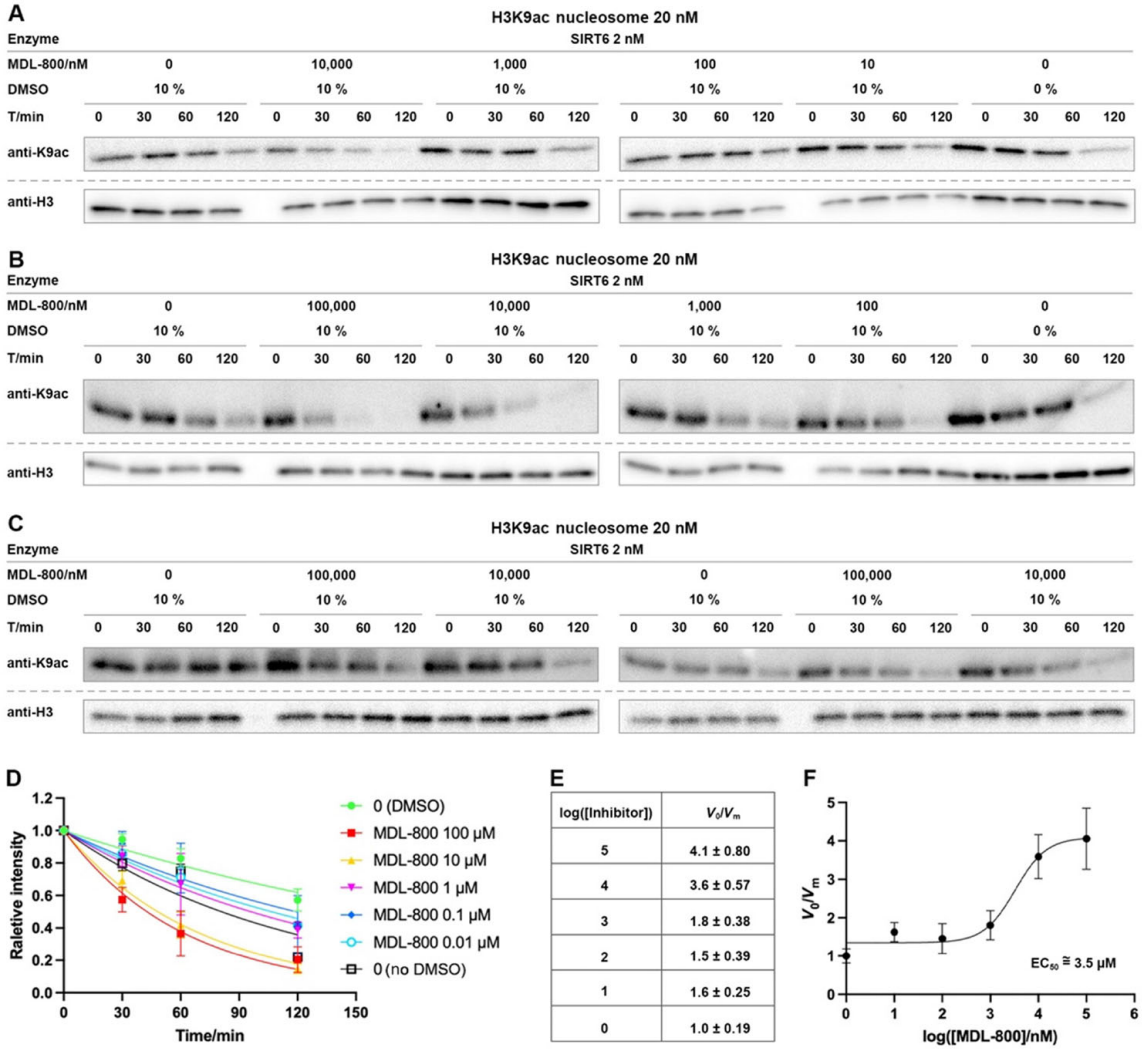
Activation assay of SIRT6 deacetylase activity by MDL‐800. (**A‐C**) Immunoblots showing time‐dependent deacetylation of H3K9ac nucleosomes (20 nM) by SIRT6 (2 nM) in the presence of the indicated concentrations of MDL‐800 and 10% DMSO (vehicle control) or no DMSO. Anti‐H3 was used as a loading control and showed no change within each assay. (**D**) Quantification of H3K9ac signal normalized to the 0 min time point, followed by fitting to a first‐order decay curve to determine *V*_0_/*V*_m_. Here, *V*_m_ represents the maximum rate within a series of inhibitor assays, typically corresponding to the reaction without inhibitor but with DMSO serving as the control. (**E**) Table of *V*₀/*V*_m_ values ± SEM for each MDL‐800 concentration. SEM was calculated using the error‐propagation formula σ(*z*) = *Z* × [(σ_*x*_/*X*)² + (σ*ᵧ*/*Y*)²]^1/2^. (**F**) Dose‐response analysis of MDL‐800 activation, with *V*_0_/*V*_m_ values plotted against log_10_[MDL‐800] and fitted to a sigmoidal dose‐response curve.

Quantify histone acetylation band intensity using ImageJ, normalize to the time 0 signal, and fit to a first‐order decay curve to obtain the *V*/[E] value for each assay. The final *V*/[E] ± SEM for MDL‐800 at 100, 10, 1, 0.1, and 0.01 µM was calculated as described above and then divided by the control *V*/[E] ± SEM for MDL‐800 = 0 (DMSO), with SEM values determined by error propagation. Curve fitting was performed using the “log(activator) vs. response, variable slope (four parameters)” model to determine the EC_50_ (3.5 ± 0.3 µM; Fig. 6; Wang et al., 2023b).

### Author Contributions

**Zhuoyi Niu**: Writing—original draft; writing—review and editing. **Ling Zhang**: Writing—original draft; writing—review and editing. **Yuanfei Zhou**: Writing—review and editing. **Zhipeng Wang**: Conceptualization; investigation; supervision; writing—original draft; writing—review and editing.

### Conflict of Interest

The authors declare no conflict of interest.

## Data Availability

All data presented in this protocol can be found in the original research papers cited at the corresponding locations.
